# Predicting benign prostatic hyperplasia risks: model development and external validation based on three cohorts

**DOI:** 10.1186/s41256-025-00456-4

**Published:** 2025-12-17

**Authors:** Hao Zi, Yong-Bo Wang, Qiao Huang, Yuan-Yuan Zhang, Fa-Zhi He, Li-Min Xing, Yan Yao, Bing-Hui Li, Li-Sha Luo, Fei Li, Shi-Di Tang, Xian-Tao Zeng, Jiao Huang

**Affiliations:** 1 Center for Evidence-Based and Translational Medicine, Zhongnan Hospital of Wuhan University, Wuhan, China; 2 Evidence-Based Medicine Center, Xiangyang No.1 People’s Hospital, Hubei University of Medicine, Xiangyang, 441000 China; 3 School of Computer Science, Wuhan University, Wuhan, China; 4 Department of Physical Examination, Integrated Chinese and Western Medicine Hospital of XiangYang (Dongfeng People’s Hospital), Xiangyang, China; 5 Department of Healthcare Management (Physical Examination Center), Zhongnan Hospital of Wuhan University, Wuhan, 430071 China; 6 Department of Urology, Zhongnan Hospital of Wuhan University, Wuhan, China

**Keywords:** Prediction model, Benign prostatic hyperplasia, Incidence, Machine learning

## Abstract

**Background:**

As benign prostatic hyperplasia (BPH) becomes increasingly prevalent, there is a growing need for simple and accurate methods to predict its risk. This study aimed to develop and validate a prediction model to identify males at high risk of developing BPH.

**Methods:**

The model was developed using data from 210,408 participants in the UK Biobank and externally validated with 5394 participants from the China Health and Retirement Longitudinal Study (CHARLS) and 294 participants from the Fengshen study. Six methods were employed to construct prediction models utilizing readily available medical characteristics at baseline. The DeLong tests were used to assess the differences in the area under the curves (AUCs). Cox regression was adopted to examine the relationships between the predictors and BPH.

**Results:**

During a median follow-up period of 13.2 years (interquartile range [IQR] 12.3–14.0), 7.0 years (IQR 6.8–7.0) and 4.0 years (IQR 2.2–5.0), 18,681 males in the UK Biobank, 309 males in the CHARLS, and 27 males in the Fengshen study developed BPH. The model developed using the LightGBM method exhibited the highest discriminative capability among the six methods. Following feature reduction based on importance ranking, a full model with 17 predictors was established for BPH prediction (AUC = 0.688 ± 0.004). Age was the most important feature that contributed to the model, with older males showing a higher hazard ratio (HR) of 1.091 (95% confidence interval [CI] 1.089–1.094) for BPH incidence. Furthermore, a final simplified model was developed using five predictors (age, hypertension time, blood glucose, urate, and serum creatinine) identified in both the CHARLS and Fengshen studies for potential clinical application. It has been transformed into a user-friendly web tool to facilitate clinical utility.

**Conclusions:**

The model, incorporating five easily accessible predictors with acceptable predictive abilities for incident BPH, can help identify individuals at high risk of BPH in the general population.

**Supplementary Information:**

The online version supplementary material available at 10.1186/s41256-025-00456-4.

## Background

Benign prostatic hyperplasia (BPH) refers to the nonmalignant growth of the prostate gland and is the most common cause of lower urinary tract symptoms (LUTS) in middle-aged and older men. Typically occurring after the age of 40, its prevalence rises substantially with age, affecting approximately 20% of males aged 51–60 years, 50% of those aged 61–70 years, and up to 83% of males aged 81–90 years [1, 2]. Recent data from the Global Burden of Disease database indicate that BPH, as a chronic non-communicable disease, had an estimated 112 million cases worldwide in 2021, making it a critical global public health concern [3]. Despite the growing impact of BPH, global efforts to devise comprehensive prevention strategies remain notably insufficient. There is an urgent need for globally coordinated efforts to integrate BPH prevention strategies into comprehensive non-communicable disease management frameworks, with the aim of effectively reducing the health burden imposed by this condition.

BPH progression is insidious and dynamic. For some individuals, symptoms of BPH may persist and worsen over time, while for others, the condition may be improved [4]. As the disease progresses, it can lead to severe complications that require surgical intervention, particularly in patients with renal insufficiency, refractory urinary retention, recurrent urinary tract infections, recurrent bladder stones, or gross hematuria due to BPH. These complications severely impact patients’ quality of life and impose a substantial financial burden [5]. The burden of BPH on healthcare systems is substantial and warrants attention. In the UK, during the 2007–2008 financial year, the estimated annual expenditure reached approximately £69 million for pharmacological management and £101 million for surgical interventions and complication management [6]. Similarly, in the United States, the annual direct healthcare costs of BPH treatment—excluding outpatient medications—reached $1.1 billion as early as 2000 [7]. Therefore, early identification of males at high risk for BPH is crucial for timely intervention to delay disease onset and progression. This not only benefits personal health management but also alleviates the burden on the healthcare system.

The diagnosis of BPH typically involves symptom assessment, physical examination, and various laboratory tests [8]. However, these tests may only be feasible after the disease has progressed to a certain extent. Developing models based on routine clinical parameters to predict the risk of developing BPH earlier could enable early intervention. While existing research on clinical prediction models for BPH has mainly focused on diagnosis, treatment, and prognosis, there is a lack of studies on predicting BPH incidence. Ren et al. developed a nomogram to predict BPH risk based on 185 patients followed up for 1.5 years, achieving a c-index of 0.857 [9]. However, this model was based on data from patients with metabolic syndrome and lacked external validation. Another study, which enrolled 3310 male participants followed up for 4 years in the China Health and Retirement Longitudinal Study (CHARLS), explored the impact of circadian rhythm syndrome (AUC = 0.574) and metabolic syndrome (AUC = 0.561) on predicting incident BPH [10]. These studies have made important explorations in predicting the incidence of BPH, but small sample sizes, short follow-up duration, and suboptimal predictive performance have limited their generalization. Moreover, these studies have predominantly focused on the Chinese Han population, despite variations in anthropometric indices among different ethnic groups. Therefore, there is a need to develop and externally validate more accurate prediction models to facilitate practical assessment for the primary prevention of BPH.

In this study, we developed and internally validated a prediction model for the 1-, 3-, 5-, and 10-year as well as lifetime risk of BPH. The model was constructedusing both traditional statistical and machine learning methods, applied to a large prospective UK Biobank cohort, 210,408 male participants with over ten years of follow-up. Furthermore, the model underwent external validation in two other independent cohorts. This study aims to (1) develop and externally validate risk prediction models to identify males at high risk of incident BPH based on common predictors that are easily accessible in clinical settings; (2) construct a user-friendly BPH prediction tool based on the best-performing model to assist estimating the probability of developing BPH, thereby facilitating the early identification of high-risk individuals. This study highlights the importance of early risk assessment for BPH prevention and management, with the objective of providing insights into the early detection of high-risk populations and the implementation of proactive preventive interventions for other chronic conditions.

## Methods

### Study participants

*UK Biobank population for model development and internal validation.* The UK Biobank is a prospective cohort that recruited over 500,000 participants aged 37–73 years between 2006 and 2010 [11]. The study received approval from the North West Multicenter Research Ethics Committee, with all participants providing written informed consent. Participants were followed up from the baseline attendance date until the first occurrence of any of the following: the incident BPH diagnosis, death, loss of follow-up, or the end of the study on July 1st, 2022. The UK Biobank approved the data used in this study under the approval number 96548. Participants were excluded from the study based on the following criteria: female sex, withdrawal of consent, a prior BPH diagnosis at baseline or within the first six months of follow-up, a diagnosis of prostate cancer prior to participation or before a BPH diagnosis during follow-up, or an age below 40 years at baseline. A total of 210,408 eligible males were included in the study. Model development and internal validation were performed using a five-fold cross-validation approach on this dataset.

*CHARLS and Fengshen participants for external model validation.* The CHARLS is a longitudinal survey designed to collect comprehensive data on middle-aged and older adults to support research and inform aging-related policy decisions in China [12]. The baseline survey, conducted in 2011, enrolled participants aged 45 years and older from 150 county-level units and 450 village-level units. Follow-up interviews were conducted in 2013, 2015, and 2018 using face-to-face computer-assisted personal interviews. Ethical approval for CHARLS was obtained from the Institutional Review Board at Peking University, with all participants providing written informed consent. The Fengshen study collected health examination data from employees of the Fengshen Xiangyang Automobile Co., Ltd., between 2017 and 2023. This data encompassed demographic information, physical examination results, complete blood counts, blood chemistry, and urinalysis. Approval for the Fengshen study was obtained from the Ethics Committee of Zhongnan Hospital of Wuhan University, with all participants providing written informed consent. For both external validation cohorts, the exclusion criteria were: female sex, a pre-existing diagnosis of BPH at baseline or within the first six months of follow-up, failure to complete any follow-up interview, and age below 40 years at baseline. A total of 5394 males from CHARLS and 294 males from the Fengshen study were eligible for the external validation of the models.

### Study variables

*Outcome variables.* In the UK Biobank, the diagnosis of BPH was established using hospital admission data from the Hospital Episode Statistics for England (up to October 31, 2022), Scottish Morbidity Record data for Scotland (up to July 31, 2021), and Patient Episode Database for Wales (up to February 28, 2018). BPH was confirmed by a medical diagnosis coded as N40 according to the International Classification of Diseases-Tenth Revision (ICD-10) [13]. In the CHARLS study, BPH diagnosis was confirmed based on an affirmative response to the question: ‘Have you been diagnosed by a doctor with prostate illness such as BPH (excluding prostate cancer)?’ [14]. In the Fengshen study, BPH diagnosis was determined by the prostate ultrasound results, with a prostate volume ≥ 30 mL indicating the presence of BPH.

*Candidate predictors.* To accurately predict the risk of BPH, this study considered predictors that are relevant and easily accessible based on current evidence [15–17]. The predictors for this study were collected at the baseline assessment. For the UK Biobank, the candidate predictors included a wide range of factors such as assessment centre, sociodemographic factors, lifestyle-related factors, psychological factors, physical measurements, health-related outcomes, complete blood counts, blood chemistry, urinalysis, and nuclear magnetic resonance (NMR) metabolomics. A total of 402 variables were identified, consisting of 374 direct and 28 derived variables (Table S1 and S2). 151 variables were left after excluding variables with a missing rate of ≥ 30%. In the CHARLS study, 26 direct variables were selected from categories like sociodemographic factors, psychological factors, health-related outcomes, blood chemistry, and physical measurements. Similarly, the Fengshen study included 32 direct variables from categories like sociodemographic factors, health-related outcomes, blood chemistry, urinalysis, and physical measurements.

### Predictor selection

Predictor selection was conducted using two methods, the least absolute shrinkage and selection operator (LASSO) and the light gradient boosting machine (LightGBM), to develop prediction models with fewer important variables. The LASSO method automatically eliminated redundant covariates by applying a penalty to the regression coefficients, thereby retaining the most significant variables in the final model [18]. The optimal regularization parameter (λ) was determined through fivefold cross-validation and the area under the receiver operating characteristic (ROC) curve (AUC) to identify the best subset of variables for predicting BPH. Application of the LASSO method resulted in the selection of 93 predictors (Fig. S1), with the top 30 predictors listed in Table S3.

The LightGBM method selected predictors through a process involving candidate feature ranking and sequential forward selection [19]. Model parameters were tuned using the Optuna method to achieve optimal performance. Features were ranked by their information gain, a measure of their ability to predict BPH incidence. The top 50 features were initially selected, and hierarchical clustering based on the Spearman rank correlation coefficients was performed to reduce information redundancy. A sequential forward selection strategy was then employed, with features within the pre-selected subset re-ranked based on a new classifier. Predictors were incrementally added based on their updated importance ranking until optimal predictive performance was achieved. The Shapley Additive exPlanations (SHAP) score was calculated to rank the feature importance and interpret the model's prediction [20]. This score help refine the predictor set from 402 to 18 features based on their importance rank in the LightGBM model (Fig. S2). A total of 17 predictors were identified for the full model, which was concurrently in the prediction factor sets selected by both the LASSO and LightGBM methods (Table S4). Cox regression was employed to examine the relationships between these 17 predictors and BPH risk. Additionally, a simplified model was constructed using five predictors all found in the UK Biobank, CHARLS and the Fengshen study to enhance its potential for clinical application (Table S5).

### Model development and comparison

This study compared the performance of models developed using both traditional statistical methods (logistic regression and Cox regression) and machine learning methods (decision tree, random forest, XGBoost, and LightGBM). The evaluation was conducted on the full models with 17 predictors to identify the best-performing method and its optimal parameters. Subsequently, a simplified model was developed using the five predictors that are all in the three cohorts based on the optimal method identified. As a sensitivity analysis, age and other four predictors randomly selected from the remaining 16 predictors were used to construct model to test whether the model performance remained consistent and comparable when using a subset of predictors compared to all 17 predictors.

Logistic regression is a classical classification algorithm that assesses the association between a categorical outcome and predictor variables using a Sigmoid function [21]. Cox regression is a survival analysis method used to analyze the relationship between predictor variables and and the time-to-event outcome. Its predictive performance over time was assessed using the time-dependent ROC analysis [22]. Among the machine learning algorithms, decision tree is a method that represents classification rules as a binary tree graph, where each leaf node represents an outcome [23]. Random forest, a model for ensemble learning, combines numerous decision trees [24]. XGBoost is another ensemble learning model that utilizes the gradient boosting algorithm to construct a robust classifier by iteratively training multiple weak classifiers and optimizing the loss function via gradient descent [25]. LightGBM could handle imbalance by adjusting various parameters to improve the model’s performance [26]. The class imbalance arises when there is a significant difference in the number of samples between the positive and negative classes.

Model performance was evaluated using metrics such as the AUC, sensitivity, specificity, positive predictive value (PPV), negative predictive value (NPV) and weighted F1-score. AUC, which quantifies discriminatory ability, ranges from 0.5 (no discrimination) to 1.0 (perfect discrimination). The Delong test was used to compare the differences in AUC between different models [27]. Additionally, calibration, which measures the agreement between predicted probabilities and actual outcomes, was evaluated using calibration plots and the Hosmer-Lemeshow goodness-of-fit test [28]. A well-calibrated model is indicated by calibration plot where the data points, usually split into deciles, closely align with the 45-degree line of perfect agreement. 

### Model external validation and webpage tool development

**Fig. 1:**
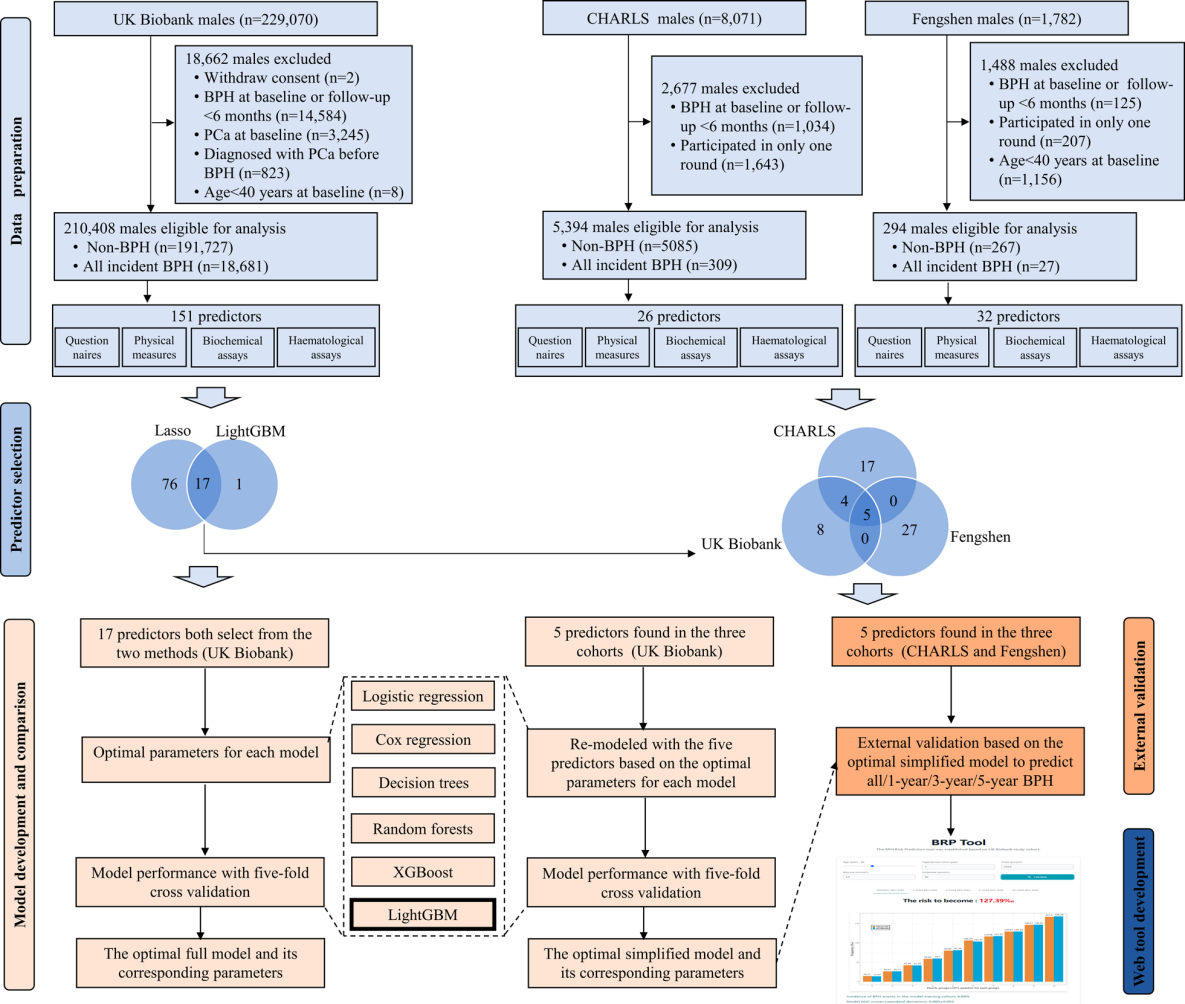
Flowchart of the study design

To enhance the generalizability of the prediction model, the simplified model based on five predictors was externally validated in the CHARLS and Fengshen study for predicting the all/1-year/3-year/5-year incident BPH. To facilitate clinical utility, the final simplified prediction model was deployed as a web application developed using the Shiny web interactive framework. Users can input individual feature values to a personalized BPH risk probability and a corresponding visualization. The summary of the study design is displayed in Fig. 1.

### Subgroup analysis

The stability and accuracy of the prediction model for predicting all/1-year/3-year/5-year/10-year incident BPH were also evaluated across different age subgroups: ≥ 45 years, those ≥ 50 years, ≥ 55 years, and ≥ 60 years.

### Statistical analysis

Variables with a high missingness of ≥ 30% were excluded from the analysis. For the remaining variables, missing data were imputed using the multivariate imputation with chained equations (MICE) algorithm. Continuous variables with normal and skewed distributions were summarized as mean ± standard deviation (SD) and median with interquartile range (IQR), respectively. Categorical variables were presented as counts with percentages. The analyses were implemented using scikit-learn library (v1.2.1), xgboost library (v2.0.3), lightgbm library (v3.3.5), optuna library (v3.2.0) and scipy library (v1.10.0) in Python version 3.10.0 (https://www.python.org) and timeROC package (v0.4), survival package (v3.4.0) and riskRegression package (v2023.3.22) in R version 4.0.1 (https://www.r-project.org).

## Results

### Demographic characteristics

The baseline characteristics of the derivation cohort (UK Biobank, n = 210,408) and two external validation cohorts (CHARLS, n = 5394; Fengshen, n =294) are presented in Table 1. In the UK Biobank, the median age was 57.0 (IQR 49.0–63.0) years, with a median urate of 350.1 (IQR 305.6–399.2) μmol/L. The median of blood glucose and serum creatinine were 4.9 (IQR 4.6–5.4) mmol/L and 79.9 (IQR 72.5–88.2) μmol/L, respectively. The median age of the CHARLS participants was similar to that of the UK Biobank at 56.0 (IQR 49.0–61.0) years. However, CHARLS participants had lower median serum creatinine (75.9, IQR 66.9–84.9 μmol/L) but higher median blood glucose (5.9, IQR 5.6–6.2 mmol/L) and urate (421.2, IQR 358.1–496.2 μmol/L) compared to those of the UK Biobank. In the Fengshen study, the median age (44.0, IQR 42.0–46.0 years) was lower than that of the UK Biobank and CHARLS. The blood glucose (5.9, IQR 5.6–6.2 mmol/L) was comparable to that of the CHARLS, while the median urate (369.0, IQR 317.0–419.5 μmol/L) was lower and the median serum creatinine (102.0, IQR 77.0–110.0 μmol/L) was higher than those of the CHARLS. During a median follow-up time of 13.2 (IQR 12.3–14.0) years in the UK Biobank, 7.0 (IQR 6.8–7.0) years in the CHARLS and 4.0 (IQR 2.2–5.0) years in the Fengshen study, incident BPH was identified in 18,681, 309, and 27 male participants, respectively. The comparisons of characteristics between individuals with BPH and without BPH in the three cohorts were presented in Table S6 and Fig. S3-S5.

**Table 1:** Baseline characteristics of the three cohorts

Participants Characteristics	UK Biobank (n=210,408)	CHARLS (n=5394)	Fengshen (n=294)
Follow-up (years), median (IQR)	13.2 (12.3–14.0)	7.0 (6.8–7.0)	4.0 (2.2–5.0)
Age (years), median (IQR)	57.0 (49.0–63.0)	56.0 (49.0–61.0)	44.0 (42.0–46.0)
BMI (kg/m^2^), median (IQR)	27.3 (25.0–30.1)	22.7 (20.5–25.2)	24.5 (22.5–26.3)
SBP (mmHg), mean±SD	142.5±18.5	129.0±21.5	128.8±15.4
DBP (mmHg), mean±SD	84.1±10.6	77.3±13.0	82.8±10.5
Hypertension time (years), median (IQR)	0.0 (0.0–0.0)	0.0 (0.0–3.0)	0.0 (0.0–0.0)
Cholesterol (mmol/L), median (IQR)	5.5 (4.7–6.2)	4.8 (4.2–5.4)	4.4 (3.9–4.8)
Urate (μmol/L), median (IQR)	350.1 (305.6–399.2)	421.2 (358.1–496.2)	369.0 (317.0–419.5)
Glucose (mmol/L), median (IQR)	4.9 (4.6–5.4)	5.9 (5.6–6.2)	5.9 (5.6–6.2)
Serum creatinine (μmol/L), median (IQR)	79.9 (72.5–88.2)	75.9 (66.9–84.9)	102.0 (77.0–110.0)
Apolipoprotein A (g/L), median (IQR)	1.4 (1.3–1.6)	/	/
Alcohol, n (%)			
Ideal	5793 (2.8)	2183 (40.5)	/
Intermediate	7331 (3.5)	399 (7.4)	/
Poor	196,508 (93.4)	1263 (23.4)	/
Missing	776 (0.4)	1549 (28.7)	/
Smoking, n (%)			
Never	102,988 (48.9)	2321 (43.0)	/
Previous	79,113 (37.6)	249 (4.6)	/
Current	26,994 (12.8)	1016 (18.8)	/
Missing	1313 (0.6)	1808 (33.5)	/
Mean corpuscular volume (fL), median (IQR)	91.4 (88.8–94.1)	/	/
IGF-1 (nmol/L), median (IQR)	21.8 (18.3–25.2)	/	/
Potassium in urine (mmol/L), median (IQR)	63.1 (42.0–88.7)	/	/
Vitamin D (nmol/L), median (IQR)	46.4 (32.0–62.0)	/	/
Phosphate (mmol/L), median (IQR)	1.1 (1.0–1.2)	/	/
Mean platelet thrombocyte volume(fL), median (IQR)	9.2 (8.5–9.9)	/	/
Creatinine in urine (μmol/L), median (IQR)	9956.0 (6213.5–14404.0)	/	/
Urea (mmol/L), median (IQR)	5.4 (4.7–6.3)	/	/
Red blood cell count (%), median (IQR)	4.8 (4.5–5.0)	/	/
HDL cholesterol (mmol/L), median (IQR)	1.2 (1.1–1.4)	/	/
Total protein (g/L), median (IQR)	72.5 (69.9–75.2)	/	/
SHBG (nmol/L), median (IQR)	36.7 (27.7–47.9)		
Basal metabolic rate (KJ), median (IQR)	7690.0 (7067.0–8393.0)	/	/
WHR, median (IQR)	0.9 (0.9–1.0)	/	/
Tense highly strung, n (%)			
No	171,241 (81.4)	/	/
Yes	31,253 (14.9)	/	/
Missing	7914 (3.8)	/	/
Worrier anxious feelings, n (%)			
No	107,458 (51.1)	/	/
Yes	96,180 (45.7)	/	/
Missing	6770 (3.2)	/	/
Miserableness, n (%)			
No	132,680 (63.1)	/	/
Yes	73,004 (34.7)	/	/
Missing	4724 (2.3)	/	/

*IQR* interquartile range, *SD* standard deviation, *BMI* body mass index, *SBP* systolic blood pressure, *DBP* diastolic blood pressure, *IGF*-1 Insulin-Like Growth Factor-1, *HDL* high-density lipoprotein, *WHR* waist to hip ratio, *SHBG* sex hormone binding globulin

### Model development and performance comparison

**Fig. 2:**
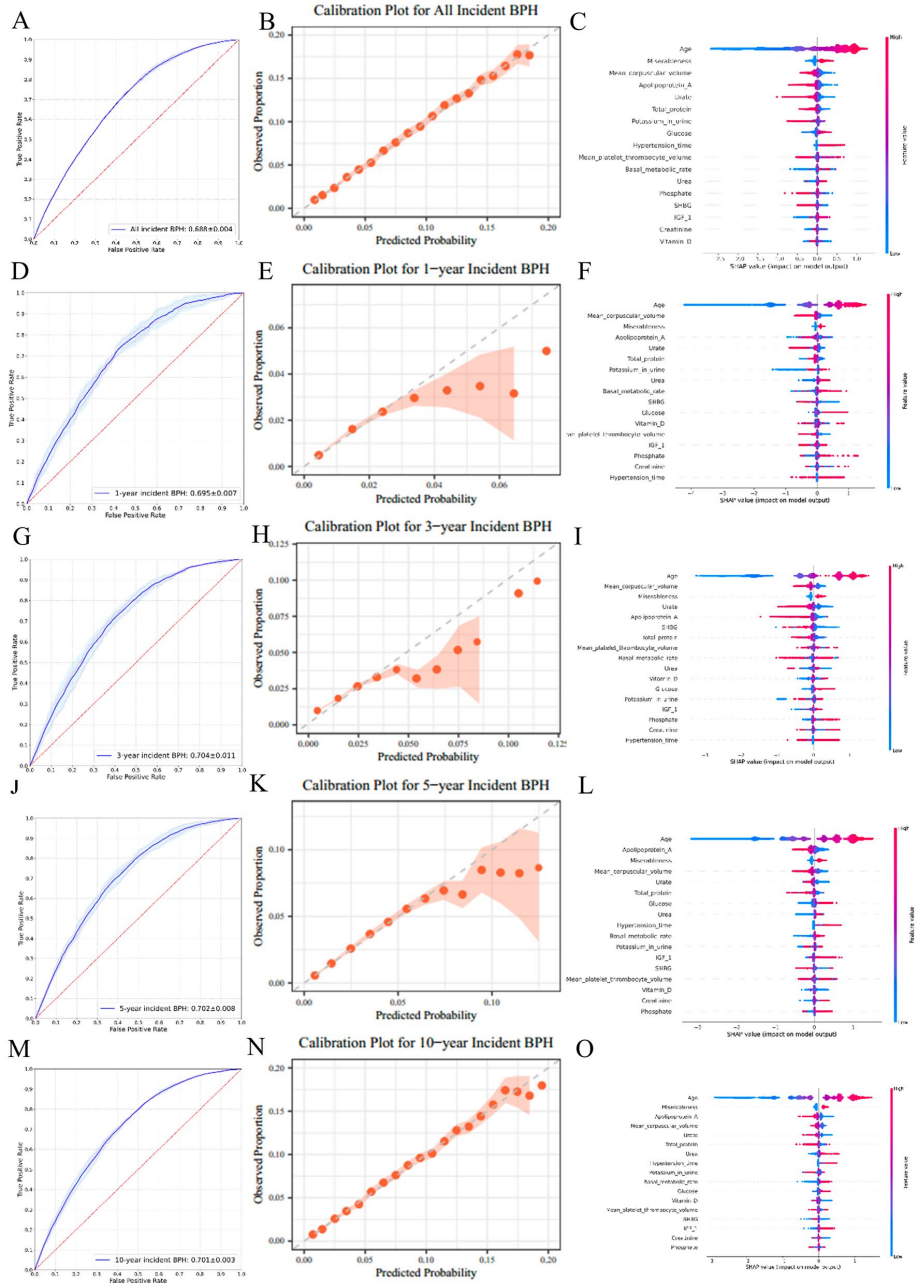
AUC plots, calibration curve plots and SHAP visualisation of modelling for the full model based on LightGBM for predicting all, 1-, 3-, 5- and 10-year BPH incidence risks in the UK biobank. **A**–**C** all incidence risks; **D**–**F** 1-year incidence risks; **G–I** 3-year incidence risks; **J**–**L** 5-year incidence risks; **M**–**O** 10-year incidence risks

Using baseline data from the UK Biobank, prediction models for incident BPH were developed with six different methods (logistic regression, Cox regression, decision tree, random forest, XGBoost, and LightGBM). For the full models incorporating 17 predictors, the AUC for predicting lifetime incident BPH ranged from 0.669 to 0.688. The LightGBM model demonstrated the highest discriminative ability, with an AUC of 0.688 (Fig. 2, Fig. S6, Table S7). Pairwise comparisons revealed statistically significant differences in AUC among all models (all *P* < 0.001, Table S8). The LightGBM model for all incident BPH showed a sensitivity of 0.754 ± 0.006, specificity of 0.525 ± 0.007, PPV of 0.134 ± 0.001, NPV of 0.956 ± 0.001 and weighted F1-score of 0.638 ± 0.005. This model also performed well in predicting incident BPH at near-term intervals, with AUCs of 0.695 ± 0.007 for 1-year, 0.704 ± 0.011 for 3-year, 0.702 ± 0.008 for 5-year and 0.701 ± 0.003 for 10-year (Table S7). The calibration plots of the LightGBM model for all/1-year/3-year/5-year/10-year incident BPH indicated a good fit between the predicted probabilities and observed proportions within all decile groups (*P* = 0.922, 0.599, 0.872, 0.995 and 0.855) (Fig. 2).

**Fig. 3:**
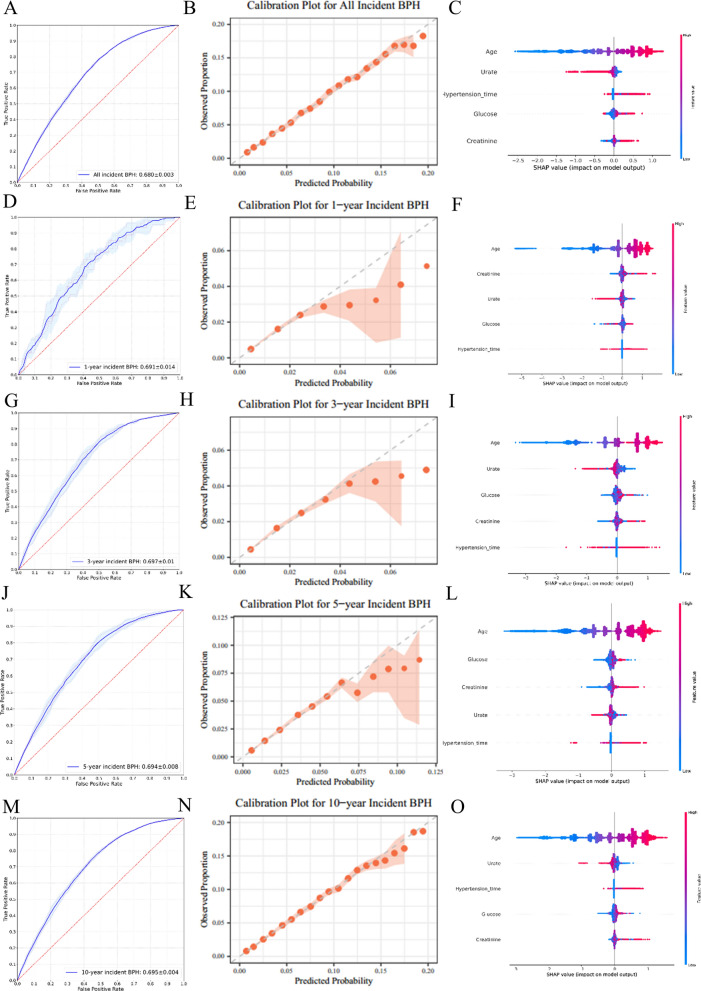
AUC plots, calibration curve plots and SHAP visualisation of modelling for the simplified model based on LightGBM for predicting all, 1-, 3-, 5- and 10-year BPH incidence risks in the UK biobank. **A**–**C** all incidence risks; **D**–**F** 1-year incidence risks; **G**–**I** 3-year incidence risks; **J**–**L** 5-year incidence risks; **M**–**O** 10-year incidence risks

The simplified models with five predictors showed AUCs ranging from 0.655 to 0.680 for all incident BPH, with the LightGBM model also achieving the highest AUC of 0.680 (Fig. 3, Fig. S7, Table S7, Table S9). The LightGBM model for all incident BPH had a sensitivity of 0.786 ± 0.010, specificity of 0.490 ± 0.007, PPV of 0.130 ± 0.001, NPV of 0.959 ± 0.001 and weighted F1-score of 0.611 ± 0.005. The model maintained robust performance across shorter prediction intervals, with AUCs of 0.691 ± 0.014 (1-year), 0.697 ± 0.010 (3-year), 0.694 ± 0.008 (5-year) and 0.695 ± 0.004 (10-year) (Table S7). The calibration plots of the LightGBM model for all/1-year/3-year/5-year/10-year incident BPH were also nicely fitted (*P* = 0.923, 0.982, 0.821, 0.884 and 0.913) that the predicted probabilities and observed actual proportions were closely matched within all decile groups (Fig. 3).

As shown in the SHAP plots, the contributions of the features to the model were exhibited in descending order. Age was the most important feature in both the full and simplified models. As detailed in Table 2 , Cox analysis of the 17 predictors revealed that advanced age was associated with an increased hazard of BPH (HR 1.091, 95%CI 1.089–1.094). And males with longer hypertension time (HR 1.034, 95%CI 1.031–1.037), higher blood glucose (HR 1.044, 95%CI 1.034–1.053), or higher serum creatinine (HR 1.002, 95%CI 1.002–1.003) had a significantly increased risk of BPH. However, higher urate was associated with a reduced likelihood of BPH (HR 0.9997, 95%CI 0.9995–0.9999). The HRs of the 17 predictors for BPH risk at 1-/3-/5-/10- year in the UK Biobank were presented in Table S10.

**Table 2:** Hazard ratios of the 17 predictors to all incident BPH in the UK Biobank

Characteristics	Univariate				Multivariate			
	HR	LL	UL	*P*	HR	LL	UL	*P*
Age	1.091	1.089	1.094	< 0.001	1.096	1.093	1.098	< 0.001
Total protein	0.968	0.964	0.972	< 0.001	0.988	0.984	0.991	< 0.001
Apolipoprotein A	0.803	0.752	0.857	< 0.001	0.679	0.634	0.727	< 0.001
Urate	0.9997	0.9995	0.9999	0.006	0.9994	0.9992	0.9996	< 0.001
Mean corpuscular volume	1.005	1.001	1.008	0.005	0.987	0.984	0.991	< 0.001
Urea	1.101	1.092	1.110	< 0.001	1.029	1.017	1.041	< 0.001
IGF-1	0.981	0.979	0.984	< 0.001	1.004	1.002	1.007	0.002
Glucose	1.044	1.034	1.053	< 0.001	1.005	1.001	1.013	0.037
Potassium in urine	0.9997	0.9993	0.9999	0.002	0.9999	0.9995	1.0003	0.682
Hypertension time	1.034	1.031	1.037	< 0.001	1.013	1.010	1.017	< 0.001
Vitamin D	1.003	1.003	1.004	< 0.001	0.9995	0.9988	1.0002	0.192
SHBG	1.007	1.007	1.008	< 0.001	0.998	0.997	0.999	< 0.001
Serum creatinine	1.002	1.002	1.003	< 0.001	1.0008	1.0001	1.0015	0.021
Phosphate	0.825	0.752	0.905	< 0.001	1.021	0.928	1.124	0.665
Miserableness								
No	Ref				Ref			
Yes	1.056	1.025	1.088	< 0.001	1.221	1.185	1.258	< 0.001
Basal metabolic rate	0.999993	0.99992	0.99995	< 0.001	1.00004	1.00003	1.00006	< 0.001
Mean platelet thrombocyte volume	0.996	0.983	0.999	0.045	0.989	0.975	1.002	0.092

*HR* Hazard ratio, *LL* lower confidence limit, *UL* upper confidence limit, *IGF*-1 Insulin-Like Growth Factor-1, *SHBG* sex hormone binding globulin

The combination of age and any other four predictors randomly selected from the remaining 16 predictors could be used to construct 1280 models. Thirty combinations were randomly selected to construct the LightGBM model for sensitivity analysis. The AUC of the models ranged from 0.677 to 0.682 for all incident BPH, demonstrating performance that was consistent and comparable with both the full 17-predictor model and the simplified 5-predictor model (Table S11).

### External validation of the final model

**Fig. 4:**
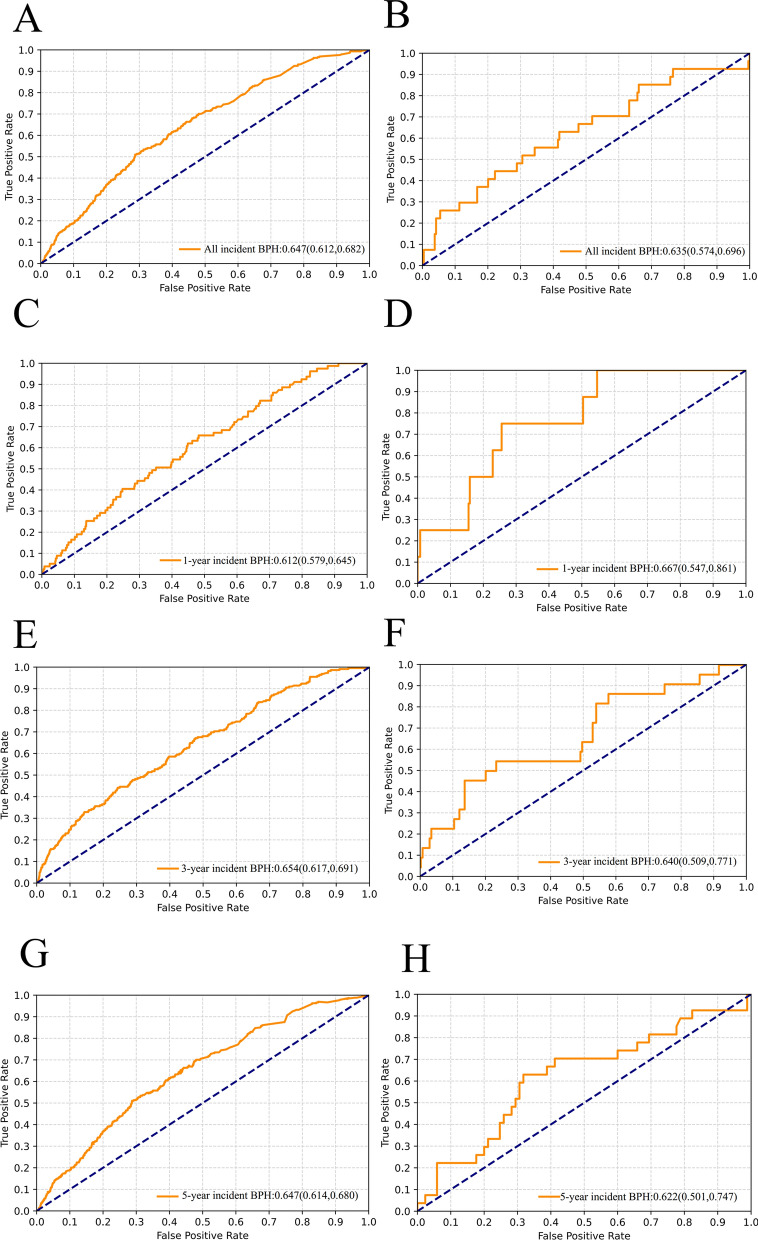
AUC plots of modelling for the simplified model based on LightGBM for predicting all, 1-, 3-, and 5-year BPH incidence risks in the CHARLS and Fengshen study. Left panels for CHARLS; Right panels for Fengshen study

The LightGBM model demonstrated similarly modest performance in the two external validation cohorts. The AUC for incident BPH was 0.647 (standard error [SE]: 0.018) in the CHARLS and 0.635 (SE: 0.031) in the Fengshen study. For near-term prediction in CHARLS, this model performed modestly in predicting incident BPH and achieved AUCs of 0.612 (SE: 0.018), 0.654 (SE: 0.019), and 0.640 (SE: 0.017) for 1-year, 3-year, and 5-year risk, respectively. Corresponding AUCs in the Fengshen study were 0.667 (SE: 0.061), 0.640 (SE: 0.066), and 0.622 (SE: 0.064) (Fig. 4 and Table S12). However, the model exhibited slightly inferior calibration for incident BPH during follow-up in the two external validation cohorts compared to the derivation cohort (Fig. S8). This disparity was primarily due to smaller number of target cases, resulting in the model's overall predicted probability being higher than the observed proportion.

### Convenient application for clinical utility

The LightGBM model has been integrated into a web application to facilitate its clinical utility, as illustrated in Fig. S9. Users can input the actual values of the five required features in the left panel to generate a personalized prediction of BPH risk, which is displayed in the red-marked right panel. The interface also includes a calibration plot showing the agreement between predicted and observed risks across deciles. The web application is available online at https://kud.whuznhmedj.com/brp.

### Subgroup analysis

The subgroup analysis showed poorer performance of the models based on the 17 predictors for predicting all/1-year/3-year/5-year/10-year incident BPH in different age ranges of males in the UK Biobank. Specifically, the AUC for predicting all incident BPH decreased to 0.688 ± 0.002 (≥45 years), 0.654 ± 0.006 (≥50 years), 0.622 ± 0.008 (≥55 years) and 0.602 ± 0.007 (≥60 years) (Table S13). Similar results were found for the models based on the five predictors, which all existed in the three cohorts (Table S14).

## Discussion

In this study, we developed and validated a prediction model for BPH risk using the LightGBM method. The model exhibited accuracy in both the derivation and validation cohorts, comprising five easily accessible and commonly available clinical predictors. It achieved AUCs of approximately 0.7 for predicting BPH incidence at 1-, 3-, 5- and longer years. Our results indicate that the proposed LightGBM model, with its satisfactory prediction accuracy and reliability, could be a practical tool for identifying high-risk populations for BPH to guide targetedg preventive strategies. This model is designed as an early risk stratification tool—not a BPH diagnostic tool— for asymptomatic middle-aged males (45–60 years) who do not undergo routine BPH screening. It aims to fill a gap in current clinical practice by providing a simple, non-invasive method for preliminary BPH risk assessment in primary care settings, thereby facilitating earlier identification of at-risk individuals. Although the model’s discriminative performance aligns with that reported in existing BPH prediction literature, its moderate AUC indicates that it should complement, not replace, clinical decision-making. As a preliminary screening aid, it is essential that individuals identified as high-risk by the model undergo further tests—such as prostate-specific antigen (PSA) detection and international prostate symptom score (IPSS) assessment—to verify their risk status and facilitate appropriate clinical management. This integrated approach helps balance screening accessibility with diagnostic reliability, ensuring that early detection leads to timely and informed patient care.

### The possible mechanisms of predictive factors

The prediction factors included in the model were age, hypertension time, blood glucose, urate, and serum creatinine, with age being identified as the most important feature. Age played a significant non-modifiable role as a risk factor in the development and progression of BPH. The other four factors were modifiable factors, underscoring the potential role of lifestyle modifications and clinical interventions in reducing BPH risk. The likelihood of histological BPH increases with age, typically manifesting after 40 years of age. Symptoms of BPH tend to worsen with age, often resulting in related complications [29]. Previous research has shown that the average prostate volume in BPH patients below 40 years old was 26.41 ml, rising significantly to 43.18 ml in patients aged over 70 years. A nomogram developed using a linear regression model based on age, height, and BMI predicted a prostate volume > 30 ml with an AUC value of 0.659 [30]. Hypertension has been found to have a positive association with BPH symptoms, independent of age. This might be due to shared pathophysiological mechanisms between the two conditions, such as increased sympathetic activity [31]. A case–control study revealed a dose–response relationship between diastolic blood pressure levels and BPH among elderly rural residents in China (OR = 1.10) [32]. While few studies have explored the relationship between the duration of hypertension and BPH, our study suggests that hypertension duration is a predictive factor for BPH incidence. It might be because long-term hypertension could affect overall health, which in turn might indirectly impact prostate health. Additionally, the management of hypertension, including specific medications or associated comorbidities, may also contribute to BPH risk. All these require further scientific validation. Our results were consistent with previous studies reporting an association between hyperglycemia and BPH risk [33]. Elevated glucose might contribute to BPH pathogenesis by downregulating the expression of pyruvate dehydrogenase kinase 4 (PDK4) and meiotic recombination 11 (MRE11) [34, 35]. Higher urate levels were associated with a reduced likelihood of LUTS and BPH in the Korean and U.S. populations, respectively [36, 37]. Our study also demonstrates a negative correlation between urate levels and BPH incidence, but the underlying mechanism requires further elucidation. The consistency of our findings with prior research provided additional validation for the efficacy of our model.

### Clinical implications and strengths of the model

With the increasing emphasis on public health, physical examinations have become a fundamental component of personal health management. However, BPH is often diagnosed only after the manifestation of clinical symptoms, as routine physical examinations generally lack dedicated tools for the early identification of individuals at risk for BPH. To bridge this gap, we developed and validated the aforementioned LightGBM-based prediction model that utilizes routinely accessible clinical variables, thereby enhancing its applicability in primary care [38].

BPH can progress to severe complications requiring surgical treatment. Consequently, early identification and management of BPH are critical for preventing such outcomes. The Fengshen validation cohort comprises a relatively younger population, highlighting the importance and practical value of early BPH prediction and prevention in this demographic [39]. The proposed model could be easily integrated into existing healthcare practices, facilitating early identification of individuals at risk for BPH and potentially leading to earlier intervention and better management of BPH.

Machine learning methods are particularly adept at identifying complex relationships within large datasets and have been widely utilized in the development of disease prediction models [40, 41]. This study compared traditional statistical methods with machine learning methods for predicting BPH. The model developed using the LightGBM method demonstrated the best performance. Its performance in the two external validation cohorts was modest, which was likely due to the limited case number and short follow-up durations. Further validation of the model is warranted to enhance its clinical utility.

The responsible clinical implementation of our prediction tool necessitates a careful consideration of the implications of both false-positive and false-negative results. A false-positive outcome, in which an asymptomatic male is misclassified as high-risk, could lead to unnecessary anxiety and financial burden for individual. From a systems perspective, it may also result in inefficient allocation of medical resources through redundant referrals, exclusionary tests, potentially increasing wait times for others and eroding confidence in the tool. Therefore, our model is explicitly positioned as a preliminary screening tool. All high-risk predictions must be interpreted by healthcare providers within the context of the patient’s complete clinical profile before initiating further action. In contrast, false-negative results—where males with truly high BPH risk are incorrectly categorized as low-risk—could foster a false sense of security, potentially leading to delayed BPH detection and intervention. To mitigate this, we emphasize that the model is not a substitute for routine preventive care. Individuals classified as low-risk are still advised to undergo annual follow-up screenings to ensure that any evolving risk is captured in subsequent evaluations.

### Implications for global public health policy

Our findings have significant implications for health policy at the individual, healthcare institutional, national, and global levels. At the individual level, the tool (available at https://kud.whuznhmedj.com/brp) developed in this study can be deployed via a mobile APP, providing a convenient and widely accessible platform for personal health management. After undergoing relevant physical examinations, individuals can input their test results into the APP to obtain an immediate assessment of their risk of developing BPH. Over time, the APP can generate a continuous BPH risk trend curve based on temporal test results, enabling users to monitor dynamic fluctuations in their BPH risk. If the trend curve indicates a sustained increase, the APP will issue an early warning, prompting individuals to implement lifestyle modifications, such as dietary adjustments, enhanced physical activity, and better sleep practices, to improve their health and mitigate BPH risk [42].

At the healthcare institutional level, the tool can be seamlessly integrated into existing public health service systems, such as Hospital Information System. When a patient undergoes relevant examination, the system will automatically capture relevant test results and calculate the patient’s BPH risk score. The identification of a high-risk score will trigger an automatic alert, facilitating timely and accurate referrals to urology specialists for further assessment and intervention. This process not only improves the efficiency of early BPH screening and intervention but also minimizes the risk of missed diagnoses or treatment delays due to human negligence [43].

At the national level, the tool can be applied to large-scale population survey data to estimate the burden of BPH risk across regions, provinces, or the entire country. Comprehensive analysis of these data can delineate the distribution of BPH risk across different regions, age groups, and living environments. These insights would enable health authorities to develop evidence-based strategies for the precise allocation of healthcare resources. For example, in regions with a high prevalence of BPH risk, targeted investments can be directed toward expanding urological care capacity. This may include the establishment of additional specialized clinics, recruitment of healthcare professionals, and procurement of advanced diagnostic equipment [44]. Concurrently, large-scale health education campaigns can be implemented to raise public awareness and promote preventive measures against BPH [45].

At the global level, the BPH risk prediction tool holds profound strategic value. The incidence and disease progression of BPH may vary across different populations and geographic regions. As current evidence does not indicate a genetic association with BPH, the prediction model—which incorporates characteristics of different populations and has been externally validated— can provide relatively accurate BPH risk assessments for men in various regions, demonstrating broad applicability. In this way, it helps reduce disparities in BPH prevention and treatment across different regions and promotes global health equity. The uneven global distribution of medical resources is a widespread issue. As a common disease, BPH diagnosis and treatment require substantial medical resources. The risk prediction model developed in this study allows healthcare institutions to optimize the allocation of limited resources by proactively identifying high-risk individuals, allowing for targeted interventions for those most in need.

## Limitations

This study had several limitations. Firstly, several potentially useful predictors for BPH, including serum PSA, prostatic acid phosphatase, and the IPSS, were excluded from model development due to their unavailability in general aging cohorts like UKB and CHARLS. Therefore, we focused on developing a prediction model using routinely available clinical indicators from health examinations. The integration of these prostate-specific markers remains a priority for our future research. Secondly, the generalizability of our findings may be limited by the relatively small sample sizes, short follow-up durations, and inconsistent outcome measurement methods in the two external validation cohorts. To mitigate this, we selected CHARLS—the most comprehensive longitudinal dataset in China, characterized by a large sample size, comprehensive measurements, high-quality data, and long follow-up duration—as our primary validation cohort supplemented by a local cohort to enhance the robustness of our validation. Lastly, it is important to recognize the limitations of machine learning algorithms. Although clinically relevant factors associated with BPH were considered during feature selection, the final predictor set was determined primarily by data-driven approaches aimed at optimizing model performance. This may introduce uncertainties regarding the model's interpretability and generalizability in diverse clinical practice. Despite these limitations, the study provides a valuable new tool for BPH risk prediction. Future studies are needed to further validate the model and enhance its utility in clinical practice.

## Conclusions

This study has successfully developed and validated a risk prediction tool for BPH targeting males aged 45 years and above, as well as their primary care providers. The final LightGBM model demonstrated good predictive performance for BPH in both internal and external validations. The model incorporates easily accessible variables that can be self-reportedor obtained from routine health examinations, which are integrated into a user-friendly online calculator, available at BRP Tool. The implementation of this tool is expected to facilitate risk-based early intervention and improved BPH management, thereby potentially reducing the disease burden of BPH in middle-aged and older male populations.

## Supplementary Information

Below is the link to the electronic supplementary material.

Supplementary material 1 (docx 2874 KB)

## Data Availability

To access to the UK Biobank resource, researchers can visit the website (https://www.ukbiobank.ac.uk/enable-your-research/apply-for-access) to submit an application requesting specific data fields. Access will be granted upon approval from the UK Biobank management team and payment of applicable fees. The CHARLS resource is freely available to the public. Researchers can apply directly for data access via the website (http://charls.pku.edu.cn/). Data from the Fengshen resource can be obtained from the corresponding author upon reasonable request.
