# Supplementary material for: Predicting benign prostatic hyperplasia risks: model development and external validation based on three cohorts
Source: Glob Health Res Policy. 2025 Dec 17;10:67. doi: 10.1186/s41256-025-00456-4 (PMC12709829; doi:10.1186/s41256-025-00456-4)
Supplement: Supplementary file 1 — Supplementary material 1 (docx 2874 KB) [file 41256_2025_456_MOESM1_ESM.docx]

Predicting benign prostatic hyperplasia risks: Model development and external validation based on three cohorts

Hao Zi^1,2†^, Yong-Bo Wang^1†^, Qiao Huang^1^, Yuan-Yuan Zhang^1^, Fa-Zhi He^3^, Li-Min Xing^4^, Yan Yao^4^, Bing-Hui Li^1,5^, Li-Sha Luo^1^, Fei Li^1,6^, Shi-Di Tang^1,6^, Xian-Tao Zeng^1,6*^, Jiao Huang^1*^

Supplementary Material

**Table S1.** List of 374 direct variables in the UK biobank.

**Table S2.** List of 28 derived variables in the UK biobank.

**Table S3.** Predictors screened by LASSO in UK biobank (top 30).

**Table S4.** 17 predictors selected for the full model.

**Table S5.** Five predictors selected for the simplified model.

**Table S6.** Population characteristics of BPH and non-BPH in the three cohorts.

**Table S7.** The performance of traditional statistical models and machine learning models for predicting BPH risk in the UK biobank.

**Table S8.** The Z score of Delong test for the comparison of the AUCs of 6 prediction models with 17 variables.

**Table S9.** The Z score of Delong test for the comparison of the AUCs of 6 prediction models with 5 variables.

**Table S10.** Hazards ratios of the 17 predictors to BPH at different timelines in the UK Biobank.

**Table S11.** Sensitivity analyses for the performance of LightGBM model based on 30 combinations of age and other four predictors.

**Table S12.** External validation based on LightGBM in the CHARLS and Fengshen cohorts.

**Table S13.** The performance of LightGBM for predicting BPH risk in the UK biobank in different age ranges with 17 variables.

**Table S14.** The performance of LightGBM for predicting BPH risk in the UK biobank in different age ranges with 5 variables.

**Fig. S1.** Predictors screened by LASSO in the UK biobank.

(A) Predictor screening process; (B) Log lambda corresponds to the best AUC and the number of predictors of non-zero coefficients.

**Fig. S2.** Predictors screened by Light GBM in UK biobank.

(A) Heatmap of correlations between predictors; (B) Hierarchical clustering based on correlation analysis; (C) Sequential forward selection for predictor screening.

**Fig. S3.** Violin plots of 17 variables between participants with BPH and without BPH in the UK Biobank.

**Fig. S4.** Violin plots of 5 variables between participants with BPH and without BPH in the CHARLS.

**Fig. S5.** Violin plots of 5 variables between participants with BPH and without BPH in the Fengshen study.

**Fig. S6.** AUC plots of the full model based on different methods on all incident BPH in the UK biobank.

(A) Cox regression; (B) Logistic regression; (C) Decision tree; (D) Random forest; (E) XGBoost; (F) LightGBM.

**Fig. S7.** AUC plots of the simplified model based on different methods on all incident BPH in the UK biobank.

(A) Cox regression; (B) Logistic regression; (C) Decision tree; (D) Random forest; (E) XGBoost; (F) LightGBM.

**Fig. S8.** Calibration curve plots of modelling for the simplified model based on LightGBM for predicting all, 1-year, 3-, and 5-year incidence risks in the CHARLS and Fengshen study.

Left panels for CHARLS; Right panels for Fengshen study.

**Fig. S9.** A stylized representation of the tool.

Table S1. List of 374 direct variables in the UK biobank.

| **Main category** | **Subcategory** | **Field ID** |
| --- | --- | --- |
| **Assessment centre (n=1)** | Recruitment (n=1) | 54 |
| **Population characteristic (n=6)** | Baseline characteristics (n=6) | 738, 6138, 6142, 21000, 21022, 22189 |
| **Touchscreen questionnaires (n=37)** | Lifestyle and environment (n=24) | 884, 894, 904, 914, 1289, 1299, 1309, 1319, 1329, 1339, 1349, 1359, 1369, 1379, 1389, 1408, 1418, 1438, 1448, 1458, 1468, 6144, 20116, 20117 |
|  | Psychosocial factors (n=13) | 1920, 1930, 1940, 1950, 1960, 1970, 1980, 1990, 2000, 2010, 2020, 2030, 2040 |
| **Physical measures (n=11)** | Anthropometry (n=9) | 48, 21001, 23099, 23105, 23111, 23115, 23119, 23123, 23127 |
|  | Blood pressure (n=2) | 4079, 4080 |
| **Health-related outcomes (n=4)** | First occurrences (n=4) | 130706, 130708, 130714, 130814 |
| **Urine assays (n=4)** | Urine biochemistry (n=4) | 30500, 30510, 30520, 30530 |
| **Blood assays (n=311)** | Blood count (n=59) | 30000, 30010, 30020, 30030, 30040, 30050, 30060, 30070, 30080, 30090, 30100, 30110, 30120, 30130, 30140, 30150, 30160, 30170, 30180, 30190, 30200, 30210, 30220, 30230, 30240, 30250, 30260, 30270, 30280, 30290, 30300, 30600, 30610, 30620, 30630, 30640, 30650, 30660, 30670, 30680, 30690, 30700, 30710, 30720, 30730, 30740, 30750, 30760, 30770, 30780, 30790, 30810, 30830, 30840, 30850, 30860, 30870, 30880, 30890 |
|  | Blood biochemistry (n=3) | 30740, 30760, 30870 |
|  | NMR metabolomics (n=249) | 23400, 23401, 23402, 23403, 23404, 23405, 23406, 23407, 23408, 23409, 23410, 23411, 23412, 23413, 23414, 23415, 23416, 23417, 23418, 23419, 23420, 23421, 23422, 23423, 23424, 23425, 23426, 23427, 23428, 23429, 23430, 23431, 23432, 23433, 23434, 23435, 23436, 23437, 23438, 23439, 23440, 23441, 23442, 23443, 23444, 23445, 23446, 23447, 23448, 23449, 23450, 23451, 23452, 23453, 23454, 23455, 23456, 23457, 23458, 23459, 23460, 23461, 23462, 23463, 23464, 23465, 23466, 23467, 23468, 23469, 23470, 23471, 23472, 23473, 23474, 23475, 23476, 23477, 23478, 23479, 23480, 23481, 23482, 23483, 23484, 23485, 23486, 23487, 23488, 23489, 23490, 23491, 23492, 23493, 23494, 23495, 23496, 23497, 23498, 23499, 23500, 23501, 23502, 23503, 23504, 23505, 23506, 23507, 23508, 23509, 23510, 23511, 23512, 23513, 23514, 23515, 23516, 23517, 23518, 23519, 23520, 23521, 23522, 23523, 23524, 23525, 23526, 23527, 23528, 23529, 23530, 23531, 23532, 23533, 23534, 23535, 23536, 23537, 23538, 23539, 23540, 23541, 23542, 23543, 23544, 23545, 23546, 23547, 23548, 23549, 23550, 23551, 23552, 23553, 23554, 23555, 23556, 23557, 23558, 23559, 23560, 23561, 23562, 23563, 23564, 23565, 23566, 23567, 23568, 23569, 23570, 23571, 23572, 23573, 23574, 23575, 23576, 23577, 23578, 23579, 23580, 23581, 23582, 23583, 23584, 23585, 23586, 23587, 23588, 23589, 23590, 23591, 23592, 23593, 23594, 23595, 23596, 23597, 23598, 23599, 23600, 23601, 23602, 23603, 23604, 23605, 23606, 23607, 23608, 23609, 23610, 23611, 23612, 23613, 23614, 23615, 23616, 23617, 23618, 23619, 23620, 23621, 23622, 23623, 23624, 23625, 23626, 23627, 23628, 23629, 23630, 23631, 23632, 23633, 23634, 23635, 23636, 23637, 23638, 23639, 23640, 23641, 23642, 23643, 23644, 23645, 23646, 23647, 23648 |

Table S2. List of 28 derived variables in the UK biobank.

| **Variable** | **Type** | **Definition** |
| --- | --- | --- |
| **WHR** | Continuous | waist-to-hip ratio |
| **WHtR** | Continuous | waist-to-height ratio |
| **Visceral adiposity index** | Continuous | [Waist circumference/(39.68+(1.88×BMI))] × (Triglycerides/1.03) × (1.31/HDL cholesterol) |
| **Cardiometabolic index** | Continuous | WHtR × (Triglycerides/HDL cholesterol) |
| **Lipid accumulation product index** | Continuous | (Waist circumference–65) ×Triglycerides |
| **Fruit** | Categorical | Fresh fruit and Dried fruit  0= Unhealthy (<3 servings/day)  1= Healthy (≥3 servings/day) |
| **Vegetable** | Categorical | Cooked vegetable Sand Salad / raw vegetable  0= Unhealthy (<3 servings/day)  1= Healthy (≥3 servings/day) |
| **Fish** | Categorical | Oily fish and Non-oily fish  0= Unhealthy (<2 servings/week)  1= Healthy (≥2 servings/week) |
| **Dairy** | Categorical | Cheese and Milk  0= Unhealthy (<2.5 servings/week)  1= Healthy (≥2.5 servings/week) |
| **Refined grains** | Categorical | Wholemeal/wholegrain bread, bran, oat, and muesli cereal  0= Unhealthy (>1.5 servings/week)  1= Healthy (≤1.5 servings/week) |
| **Processed meats** | Categorical | Processed meat and Age when last ate any kind of meat, 0 if indicated having never eaten meat  0= Unhealthy (>1 servings/week)  1= Healthy (≤1 servings/week) |
| **Unprocessed meats** | Categorical | Poultry, Beef, Lamb, Pork and Age when last ate any kind of meat, 0 if indicated having never eaten meat  0= Unhealthy (>1 servings/week)  1= Healthy (≤1.5 servings/week) |
| **Sugar-sweetened beverages** | Categorical | Never eats sugar or foods/drinks containing sugar  0= Unhealthy (>1 servings/week)  1= Healthy (≤1 servings/week) |
| **Diet patterns** | Categorical | Fruit, Vegetable, Fish, Dairy, Refined grains, Processed meats, Unprocessed meats, and Sugar-sweetened beverages  1= Unfavorable (one or fewer healthy factor)  2= Intermediate (two healthy factors)  3= Favorable (at least three healthy factors) |
| **Physical activity** | Categorical | Moderate physical activity 10+ minutes and Vigorous physical activity 10+ minutes  0= Unhealthy (moderate activity <5 days a week or vigorous activity <3 days a week)  1= Healthy (moderate activity ≥5 days a week or vigorous activity ≥3 days a week) |
| **lifestyle (categorical)** | Categorical | Smoking, BMI, Physical activity, and Diet patterns  1= Poor (≤1 healthy lifestyle factors)  2= Intermediate (2 healthy lifestyle factors)  3= Ideal (≥3 healthy lifestyle factors) |
| **Sleep index total** | Continuous | The low‐risk categories of each component were no self‐reported snoring, early chronotype (“morning” or “morning than evening”), no frequent daytime sleepiness (“never/rarely” or “sometimes”), normal sleep duration (7–8 h/day), reported never or rarely having insomnia symptoms, and getting up easy in morning (“fairly easy” or “very easy”). For each sleep behavior, the participant received a score of 1 if he or she was classified as the low‐risk group or 0 if otherwise as the high‐risk group. All component scores were summed to obtain a continuous sleep index ranging from 0 (worst) to 6 (best), with a higher index indicating a general better sleep quality. |
| **Sleep index** | Categorical | Sleep index total  1= Ideal (5-6)  2= Intermediate (3-4)  3= Poor (0-2) |
| **Sedentary total** | Continuous | Time spent on TV viewing and leisure-time computer use (both of which are non-occupational) on a typical day was each reported in 1-h increments |
| **Sedentary** | Categorical | Three categories of TV viewing and computer use were generated: ≤1h/day, 2–3h/day and ≥4h/day  1= Ideal (≤1h/day)  2= Intermediate (2–3h/day)  3= Poor (≥4h/day) |
| **Mental health** | Continuous | Mental health questions: Mood swings, Miserableness, Irritability, Sensitivity / hurt feelings, Fed-up feelings, Nervous feelings, Worrier / anxious feelings, Tense / 'highly strung', Worry too long after embarrassment, Suffer from 'nerves', Loneliness/ isolation, Guilty feelings, and Risk taking.  Total mental health complaints reported by adding up participant’s answers to the 13 UKB mental health questions described above, where higher numbers represent more mental health-related symptomatology. |
| **Frailty phenotype total** | Continuous | The criteria are derived from the self-reported variables entered by participants including: weight loss, hand grip strength, slow walking pace and low physical activity. |
| **Frailty phenotype** | Categorical | Participants will be classified as robust (met none of the frailty criteria), pre-frail (met one or two of the criteria) or frail (met three or more of the criteria)  1= Robust (0)  2= Pre-frail (1-2)  3= Frail (≥3) |
| **Hypertension_Inci** | Categorical | Date first reported essential hypertension, hypertensive heart disease, hypertensive renal disease, hypertensive heart and renal disease, secondary hypertension  0= No  1= Yes |
| **Hypertension time** | Continuous | Baseline to first report of hypertension |
| **Type_2_diabetes_inci** | Categorical | Date first reported non-insulin-dependent diabetes mellitus and unspecified diabetes mellitus. First occurrence for  exclusion type 1 diabetes: Date first reported insulin-dependent diabetes mellitus.  0= No  1= Yes |
| **Type_2_diabetes_time** | Continuous | Baseline to first report of Type 2 diabetes |
| **Dyslipidemia_Inci** | Categorical | Date first reported disorders of lipoprotein metabolism and  other lipidemia  0= No  1= Yes |

Table S3. Predictors screened by LASSO in UK biobank (top 30).

| **Rank** | **Predictors** | **Coefficient** |
| --- | --- | --- |
| **1** | Age | 0.051717529 |
| **2** | Apolipoprotein_A | -0.005517929 |
| **3** | SHBG | -0.004766942 |
| **4** | Red_blood_cell_erythrocyte_count | 0.004701606 |
| **5** | Household_income | -0.004279341 |
| **6** | Urea | 0.004225137 |
| **7** | Total_protein | -0.004218364 |
| **8** | Urate | -0.003939113 |
| **9** | Tense_highly_strung | 0.003750755 |
| **10** | Testosterone | 0.003738372 |
| **11** | Sleep_index_total | -0.00331566 |
| **12** | Miserableness | 0.00303646 |
| **13** | Hypertension_Inci | 0.00300227 |
| **14** | Platelet_count | 0.002843927 |
| **15** | Frailty_phenotype_total | 0.002763542 |
| **16** | Glucose | 0.002607629 |
| **17** | Dyslipidemia_Inci | 0.002548897 |
| **18** | Apolipoprotein_B | -0.00232981 |
| **19** | Hand_grip_strength | 0.002108867 |
| **20** | College_categorical | -0.001986365 |
| **21** | Mean_reticulocyte_volume | -0.001980279 |
| **22** | Calcium | 0.001930793 |
| **23** | Hypertension_time | 0.00188812 |
| **24** | Worry_too_long_after_embarrassment | 0.001804715 |
| **25** | Type_2_diabetes_inci | -0.001783242 |
| **26** | Lymphocyte_percentage | -0.001773474 |
| **27** | Worrier_anxious_feelings | 0.001749151 |
| **28** | Basal_metabolic_rate | 0.001709368 |
| **29** | IGF_1 | 0.001508098 |
| **30** | Sedentary | 0.001494796 |

Table S4. 17 predictors selected for the full model.

| **Predictors** | **Description** | **Category** | **Type** | **Code** |
| --- | --- | --- | --- | --- |
| **Age** | Age at recruitment | Population characteristic | Continuous | NA |
| **Total_protein** | Total protein | Blood assays | Continuous | NA |
| **Apolipoprotein_A** | Apolipoprotein A | Blood assays | Continuous | NA |
| **Urate** | Urate | Blood assays | Continuous | NA |
| **Mean_corpuscular_volume** | Mean corpuscular volume | Blood assays | Continuous | NA |
| **Urea** | Urea | Blood assays | Continuous | NA |
| **IGF_1** | IGF-1 | Blood assays | Continuous | NA |
| **Glucose** | Glucose | Blood assays | Continuous | NA |
| **Potassium_in_urine** | Potassium in urine | Urine assays | Continuous | NA |
| **Hypertension_time** | Times from diagnosis of hypertension | Self-generated variables | Continuous | NA |
| **Vitamin_D** | Vitamin D | Blood assays | Continuous | NA |
| **SHBG** | Sex hormone-binding globulin | Blood assays | Continuous | NA |
| **Creatinine** | Creatinine | Blood assays | Continuous | NA |
| **Phosphate** | Phosphate | Blood assays | Continuous | NA |
| **Miserableness** | Miserableness | Touchscreen questionnaires | Categorical | 0= No  1= Yes |
| **Basal_metabolic_rate** | Basal metabolic rate | Physical measures | Continuous | NA |
| **Mean_platelet_thrombocyte_volume** | Mean platelet (thrombocyte) volume | Blood assays | Continuous | NA |

Table S5. Five predictors selected for the simplified model.

| **Predictors** | **Description** | **Category** | **Type** |
| --- | --- | --- | --- |
| **Age** | Age at recruitment | Population characteristic | Continuous |
| **Glucose** | Glucose | Blood assays | Continuous |
| **Urate** | Urate | Blood assays | Continuous |
| **Hypertension_time** | Times from diagnosis of hypertension | Self-generated variables | Continuous |
| **Creatinine** | Creatinine | Blood assays | Continuous |

Table S6. Population characteristics of BPH and non-BPH in the three cohorts.

| Participants Characteristics | UK Biobank | | | CHARLS | | | Fengshen | | |
| --- | --- | --- | --- | --- | --- | --- | --- | --- | --- |
|  | Healthy control (n=191,727) | Incident BPH (n=18,681) | P | Healthy control (n=5085) | Incident BPH (n=309) | P | Healthy control (n=267) | Incident BPH (n=27) | P |
| Follow-up (years), median (IQR) | 13.3 (12.5–14.0) | 7.1 (3.9–10.0) | <0.001 | 7.0 (6.9–7.0) | 2.4 (1.0–3.0) | <0.001 | 4.0 (3.0–5.0) | 2.0 (1.0–3.0) | <0.001 |
| Age (years), median (IQR) | 57.0 (49.0–63.0) | 62.0 (57.0–66.0) | <0.001 | 56.0 (49.0–61.0) | 59.0 (53.0–64.0) | <0.001 | 43.0 (42.0–46.0) | 47.0 (46.0–51.0) | <0.001 |
| BMI (kg/m^2^), median (IQR) | 27.3 (25.0–30.0) | 27.5 (25.2–30.3) | <0.001 | 22.6 (20.5–25.2) | 23.2 (20.8–26.0) | 0.232 | 24.4 (22.5–26.3) | 25.3 (22.9–26.2) | 0.516 |
| SBP (mmHg), mean±SD | 142.3 (18.4) | 144.6 (18.9) | <0.001 | 128.9 (21.5) | 129.7 (21.9) | 0.644 | 128.9 (15.2) | 127.4 (17.4) | 0.619 |
| DBP (mmHg), mean±SD | 84.1 (10.6) | 84.0 (10.5) | 0.148 | 77.3 (13.0) | 77.4 (12.8) | 0.919 | 82.6 (10.4) | 84.6 (11.3) | 0.367 |
| Hypertension time (years),  median (IQR) | 0.0 (0.0–0.0) | 0.0 (0.0–0.0) | <0.001 | 0.0 (0.0–3.0) | 2.0 (0.0–7.0) | <0.001 | 0.0 (0.0–0.0) | 0.0 (0.0–0.0) | <0.001 |
| Cholesterol (mmol/L), median (IQR) | 5.5 (4.7–6.2) | 5.3 (4.5–6.1) | <0.001 | 4.8 (4.2–5.4) | 4.8 (4.2–5.5) | 0.513 | 4.3 (4.0–4.8) | 4.7 (3.8–5.3) | 0.257 |
| Urate (μmol/L), median (IQR) | 350.2  (305.8–399.2) | 348.6  (303.4–398.5) | 0.002 | 420.7  (358.2–494.8) | 423.4  (354.8–504.8) | 0.825 | 367.0  (316.5–418.0) | 381.0  (352.0–434.5) | 0.323 |
| Glucose (mmol/L), median (IQR) | 4.9 (4.6–5.3) | 5.0 (4.6–5.4) | <0.001 | 5.7 (5.2–6.3) | 5.7 (5.3–6.4) | 0.708 | 5.9 (5.6–6.2) | 5.7 (5.6–6.0) | 0.424 |
| Serum creatinine (μmol/L), median (IQR) | 79.9 (72.4–88.1) | 80.4 (72.8–89.2) | <0.001 | 75.9 (66.9–84.9) | 74.9 (66.9–84.4) | 0.885 | 103.0  (77.0–110.0) | 96.0  (79.0–108.0) | 0.636 |
| Apolipoprotein A (g/L), median (IQR) | 1.4 (1.3–1.6) | 1.4(1.3-1.5) | <0.001 | / | / |  | / | / |  |
| Alcohol, n (%) |  |  | <0.001 |  |  | 0.861 |  |  | / |
| Ideal | 5244 (2.7) | 549 (2.9) |  | 2058 (40.5) | 125 (40.5) |  | / | / |  |
| Intermediate | 6519 (3.4) | 812 (4.3) |  | 376 (7.4) | 23 (7.4) |  | / | / |  |
| Poor | 179260 (93.5) | 17248 (92.3) |  | 1196 (23.5) | 67 (21.7) |  | / | / |  |
| Missing | 704 (0.4) | 72 (0.4) |  | 1455 (28.6) | 94 (30.4) |  | / | / |  |
| Smoking, n (%) |  |  | <0.001 |  |  | 0.058 |  |  | / |
| Never | 94617 (49.4) | 8371 (44.8) |  | 2172 (42.7) | 149 (48.2) |  | / | / |  |
| Previous | 70884 (37.0) | 8229 (44.1) |  | 235 (4.6) | 14 (4.5) |  | / | / |  |
| Current | 25068 (13.1) | 1926 (10.3) |  | 975 (19.2) | 41 (13.3) |  | / | / |  |
| Missing | 1158 (0.6) | 155 (0.8) |  | 1703 (33.5) | 105 (34.0) |  | / | / |  |
| Mean corpuscular volume (fL), median (IQR) | 91.4 (88.8–94.1) | 91.4 (88.8–94.1) | 0.219 | / | / | / | / | / | / |
| IGF-1 (nmol/L), median (IQR) | 21.9 (18.3–25.2) | 21.4 (17.8–24.7) | <0.001 | / | / | / | / | / | / |
| Potassium in urine (mmol/L), median (IQR) | 63.1 (41.9–88.8) | 63.2 (43.4–87.7) | 0.228 | / | / | / | / | / | / |
| Vitamin D (nmol/L), median (IQR) | 46.3 (31.9–61.8) | 48.4 (33.5–63.9) | <0.001 | / | / | / | / | / | / |
| Phosphate (mmol/L), median (IQR) | 1.1 (1.0–1.2) | 1.1 (1.0–1.2) | <0.001 | / | / | / | / | / | / |
| Mean platelet thrombocyte volume(fL), median (IQR) | 9.2 (8.5–9.9) | 9.2 (8.5–9.9) | 0.564 | / | / | / | / | / | / |
| Creatinine in urine (μmol/L), median (IQR) | 9978.0  (6207.0–14447.0) | 9729.0  (6262.0–13952.0) | <0.001 | / | / | / | / | / | / |
| Urea (mmol/L), median (IQR) | 5.4 (4.7–6.3) | 5.6 (4.8–6.5) | <0.001 | / | / | / | / | / | / |
| Red blood cell count (%), median (IQR) | 4.8 (4.5–5.0) | 4.7 (4.5–5.0) | 0.107 | / | / | / | / | / | / |
| HDL cholesterol (mmol/L), median (IQR) | 1.2 (1.1–1.5) | 1.2 (1.0–1.4) | <0.001 | / | / | / | / | / | / |
| Total protein (g/L), median (IQR) | 72.5 (70.0–75.3) | 72.0 (69.4–74.7) | <0.001 | / | / | / | / | / | / |
| SHBG (nmol/L), median (IQR) | 36.5 (27.5–47.6) | 38.4 (29.2–49.7) | <0.001 | / | / | / | / | / | / |
| Basal metabolic rate (KJ), median (IQR) | 7699.0  (7075.0–8401.0) | 7627.0  (7012.0–8318.0) | <0.001 | / | / | / | / | / | / |
| WHR, median, (IQR) | 0.9 (0.9–1.0) | 0.9 (0.9–1.0) | <0.001 | / | / | / | / | / | / |
| Tense highly strung, n (%) |  |  | <0.001 |  |  | / |  |  | / |
| No | 156404 (81.6) | 14837 (79.4) |  | / | / |  | / | / |  |
| Yes | 28146 (14.7) | 3107 (16.6) |  | / | / |  | / | / |  |
| Missing | 7177 (3.8) | 737 (4.0) |  | / | / |  | / | / |  |
| Worrier anxious feelings, n (%) |  |  | <0.001 |  |  | / |  |  | / |
| No | 98521(51.4) | 8937(47.9) |  | / | / |  | / | / |  |
| Yes | 87062 (45.4) | 9118 (48.8) |  | / | / |  | / | / |  |
| Missing | 6144 (3.2) | 626 (3.4) |  | / | / |  | / | / |  |
| Miserableness, n (%) |  |  | 0.001 |  |  | / |  |  | / |
| No | 121128(63.2) | 11552(61.8) |  | / | / |  | / | / |  |
| Yes | 66321 (34.6) | 6683 (35.8) |  | / | / |  | / | / |  |
| Missing | 4278 (2.2) | 446 (2.4) |  | / | / |  | / | / |  |

IQR: interquartile range; SD: standard deviation; BMI: body mass index; SBP: systolic blood pressure; DBP: diastolic blood pressure; IGF-1: Insulin-Like Growth Factor-1; HDL: high-density lipoprotein; WHR: waist to hip ratio; SHBG: sex hormone binding globulin.

Table S7. The performance of traditional statistical models and machine learning models for predicting BPH risk in the UK biobank.

| **Model** | **AUC** | **Sensitivity** | **Specificity** | **PPV** | **NPV** | **Weighted F1-score** |
| --- | --- | --- | --- | --- | --- | --- |
| **Full model*** |  |  |  |  |  |  |
| **Cox regression** | 0.685±0.011 | 0.724±0.008 | 0.541±0.008 | 0.123±0.001 | 0.953±0.001 | 0.631±0.003 |
| **Logistic regression** | 0.679±0.003 | 0.778±0.003 | 0.493±0.005 | 0.13±0.001 | 0.958±0.001 | 0.613±0.004 |
| **Decision tree** | 0.669±0.006 | 0.774±0.007 | 0.501±0.004 | 0.131±0.001 | 0.958±0.001 | 0.620±0.003 |
| **Random forest** | 0.682±0.005 | 0.746±0.005 | 0.531±0.004 | 0.134±0.001 | 0.956±0.001 | 0.643±0.003 |
| **XGBoost** | 0.685±0.004 | 0.768±0.006 | 0.488±0.007 | 0.130±0.001 | 0.959±0.001 | 0.609±0.006 |
| **LightGBM** |  |  |  |  |  |  |
| **all** | 0.688±0.004 | 0.754±0.006 | 0.525±0.007 | 0.134±0.001 | 0.956±0.001 | 0.638±0.005 |
| **1-year** | 0.695±0.007 | 0.740±0.015 | 0.561±0.008 | 0.022±0.001 | 0.994±0.001 | 0.709±0.006 |
| **3-year** | 0.704±0.011 | 0.716±0.03 | 0.593±0.010 | 0.029±0.001 | 0.992±0.001 | 0.731±0.007 |
| **5-year** | 0.702±0.008 | 0.787±0.009 | 0.521±0.005 | 0.048±0.001 | 0.987±0.001 | 0.664±0.004 |
| **10-year** | 0.701±0.003 | 0.786±0.006 | 0.514±0.005 | 0.110±0.001 | 0.969±0.001 | 0.638±0.004 |
| **Simplified model^#^** |  |  |  |  |  |  |
| **Cox regression** | 0.676±0.012 | 0.785±0.003 | 0.492±0.002 | 0.125±0.001 | 0.951±0.001 | 0.612±0.003 |
| **Logistic regression** | 0.666±0.013 | 0.774±0.006 | 0.502±0.004 | 0.131±0.001 | 0.958±0.001 | 0.62±0.003 |
| **Decision tree** | 0.655±0.012 | 0.780±0.005 | 0.494±0.006 | 0.131±0.001 | 0.958±0.001 | 0.614±0.005 |
| **Random forest** | 0.674±0.003 | 0.777±0.007 | 0.499±0.003 | 0.131±0.001 | 0.958±0.001 | 0.618±0.002 |
| **XGBoost** | 0.678±0.002 | 0.771±0.007 | 0.502±0.006 | 0.131±0.001 | 0.957±0.001 | 0.62±0.004 |
| **LightGBM** |  |  |  |  |  |  |
| **all** | 0.680±0.003 | 0.786±0.010 | 0.49±0.007 | 0.130±0.001 | 0.959±0.001 | 0.611±0.005 |
| **1-year** | 0.691±0.014 | 0.821±0.014 | 0.471±0.02 | 0.020±0.001 | 0.995±0.001 | 0.632±0.018 |
| **3-year** | 0.697±0.010 | 0.815±0.014 | 0.492±0.004 | 0.026±0.001 | 0.994±0.001 | 0.649±0.003 |
| **5-year** | 0.694±0.008 | 0.788±0.016 | 0.517±0.005 | 0.048±0.001 | 0.987±0.001 | 0.661±0.004 |
| **10-year** | 0.695±0.004 | 0.774±0.003 | 0.523±0.006 | 0.11±0.001 | 0.968±0.0 | 0.645±0.005 |

*Full model based on 17 predictors. ^#^Simplified model based on 5 predictors.

AUC: area under curve; PPV: positive predictive value; NPV: negative predictive value.

Values were presented with mean±standard deviation based on five-fold cross-validation.

Results were calculated based on the regressed predicted probabilities. Cut-offs were determined based on the achievement of the largest Youden index (YI = sensitivity + specificity - 1).

Table S8. The Z score of Delong test for the comparison of the AUCs of 6 prediction models with 17 variables.

| Model comparison | Cox regression | Logistic regression | Decision tree | Random forest | XGBoost | LightGBM |
| --- | --- | --- | --- | --- | --- | --- |
| Cox regression | / | 22.01^#^ | 11.452 | 25.531 | -26.724 | -30.313 |
| Logistic regression | <0.001* | / | 14.88 | -7.721 | -8.521 | -15.407 |
| Decision tree | <0.001 | <0.001 | / | -26.033 | -25.562 | -28.711 |
| Random forest | <0.001 | <0.001 | <0.001 | / | -2.465 | -14.487 |
| XGBoost | <0.001 | <0.001 | <0.001 | <0.001 | / | -11.258 |
| LightGBM | <0.001 | <0.001 | <0.001 | <0.001 | <0.001 | / |

^#^Z score; **P* is the result of Delong test of AUC curve based on the comparison of each prediction model.

Table S9. The Z score of Delong test for the comparison of the AUCs of 6 prediction models with 5 variables.

| Model comparison | Cox regression | Logistic regression | Decision tree | Random forest | XGBoost | LightGBM |
| --- | --- | --- | --- | --- | --- | --- |
| Cox regression | / | 18.362^#^ | 16.615 | 26.477 | -28.731 | -26.473 |
| Logistic regression | <0.001* | / | -3.729 | -15.828 | -11.406 | -13.673 |
| Decision tree | <0.001 | <0.001 | / | -19.846 | -13.242 | -17.616 |
| Random forest | <0.001 | <0.001 | <0.001 | / | -0.551 | -6.834 |
| XGBoost | <0.001 | <0.001 | <0.001 | <0.001 | / | -5.514 |
| LightGBM | <0.001 | <0.001 | <0.001 | <0.001 | <0.001 | / |

^#^Z score; **P* is the result of Delong test of AUC curve based on the comparison of each prediction model.

Table S10. Hazards ratios of the 17 predictors to BPH at different timelines in the UK Biobank.

| Characteristics | 1-year incident BPH | | | | 3-year incident BPH | | | | 5-year incident BPH | | | | 10-year incident BPH | | | |
| --- | --- | --- | --- | --- | --- | --- | --- | --- | --- | --- | --- | --- | --- | --- | --- | --- |
|  | HR | LL | UL | P | HR | LL | UL | P | HR | LL | UL | P | HR | LL | UL | P |
| Univariate |  |  |  |  |  |  |  |  |  |  |  |  |  |  |  |  |
| Age | 1.112 | 1.098 | 1.126 | <0.001 | 1.108 | 1.102 | 1.113 | <0.001 | 1.102 | 1.097 | 1.106 | <0.001 | 1.097 | 1.094 | 1.100 | <0.001 |
| Total protein | 0.964 | 0.945 | 0.984 | 0.001 | 0.961 | 0.952 | 0.969 | <0.001 | 0.962 | 0.956 | 0.968 | <0.001 | 0.966 | 0.962 | 0.970 | <0.001 |
| Apolipoprotein A | 0.791 | 0.557 | 1.124 | 0.191 | 0.786 | 0.675 | 0.915 | 0.002 | 0.709 | 0.632 | 0.795 | <0.001 | 0.803 | 0.744 | 0.866 | <0.001 |
| Urate | 1.000 | 0.998 | 1.001 | 0.464 | 0.999 | 0.999 | 1.000 | 0.006 | 0.9996 | 0.9992 | 0.9999 | 0.015 | 0.99973 | 0.99949 | 0.99996 | 0.024 |
| Mean corpuscular volume | 0.990 | 0.973 | 1.008 | 0.282 | 1.00002 | 0.99244 | 1.00766 | 0.996 | 0.997 | 0.992 | 1.003 | 0.385 | 1.006 | 1.002 | 1.010 | 0.002 |
| Urea | 1.123 | 1.082 | 1.165 | <0.001 | 1.118 | 1.099 | 1.137 | <0.001 | 1.118 | 1.103 | 1.132 | <0.001 | 1.113 | 1.103 | 1.123 | <0.001 |
| IGF-1 | 0.974 | 0.960 | 0.989 | 0.001 | 0.98 | 0.974 | 0.987 | <0.001 | 0.981 | 0.976 | 0.986 | <0.001 | 0.98 | 0.977 | 0.983 | <0.001 |
| Glucose | 1.027 | 0.977 | 1.080 | 0.296 | 1.046 | 1.025 | 1.067 | <0.001 | 1.044 | 1.029 | 1.06 | <0.001 | 1.049 | 1.038 | 1.060 | <0.001 |
| Potassium in urine | 0.999 | 0.997 | 1.002 | 0.565 | 0.99996 | 0.99896 | 1.00095 | <0.001 | 0.9999 | 0.9991 | 1.0006 | 0.755 | 0.999 | 0.999 | 1.000 | 0.190 |
| Hypertension time | 1.024 | 1.006 | 1.041 | 0.007 | 1.038 | 1.032 | 1.044 | <0.001 | 1.036 | 1.031 | 1.041 | <0.001 | 1.036 | 1.032 | 1.039 | <0.001 |
| Vitamin D | 1.003 | 0.999 | 1.007 | 0.117 | 1.004 | 1.002 | 1.005 | <0.001 | 1.004 | 1.003 | 1.005 | <0.001 | 1.003 | 1.002 | 1.004 | <0.001 |
| SHBG | 1.009 | 1.005 | 1.013 | <0.001 | 1.007 | 1.005 | 1.009 | <0.001 | 1.007 | 1.006 | 1.009 | <0.001 | 1.008 | 1.007 | 1.009 | <0.001 |
| Serum creatinine | 1.003 | 1.002 | 1.005 | <0.001 | 1.003 | 1.002 | 1.003 | <0.001 | 1.002 | 1.002 | 1.003 | <0.001 | 1.002 | 1.002 | 1.003 | <0.001 |
| Phosphate | 0.991 | 0.602 | 1.633 | 0.972 | 0.894 | 0.72 | 1.109 | 0.307 | 0.911 | 0.775 | 1.07 | 0.255 | 0.854 | 0.767 | 0.950 | 0.004 |
| Basal metabolic rate | 1.000 | 1.000 | 1.000 | 0.029 | 0.99993 | 0.9999 | 0.99996 | <0.001 | 0.99994 | 0.99991 | 0.99996 | <0.001 | 0.99997 | 0.99995 | 0.99999 | <0.001 |
| Mean platelet thrombocyte volume | 0.959 | 0.891 | 1.032 | 0.263 | 0.985 | 0.954 | 1.016 | 0.331 | 0.987 | 0.964 | 1.011 | 0.278 | 0.994 | 0.978 | 1.009 | 0.432 |
| Miserableness |  |  |  |  |  |  |  |  |  |  |  |  |  |  |  |  |
| No | Ref. |  |  |  | Ref. |  |  |  | Ref. |  |  |  | Ref. |  |  |  |
| Yes | 1.036 | 0.882 | 1.217 | 0.666 | 1.035 | 0.966 | 1.110 | 0.329 | 1.067 | 1.014 | 1.124 | 0.014 | 1.058 | 1.022 | 1.095 | 0.001 |
| Multivariable |  |  |  |  |  |  |  |  |  |  |  |  |  |  |  |  |
| Age | 1.117 | 1.102 | 1.132 | <0.001 | 1.112 | 1.106 | 1.119 | <0.001 | 1.106 | 1.102 | 1.111 | <0.001 | 1.101 | 1.098 | 1.104 | <0.001 |
| Total protein | 0.986 | 0.966 | 1.007 | 0.191 | 0.983 | 0.974 | 0.992 | 0.0002 | 0.984 | 0.978 | 0.991 | <0.001 | 0.987 | 0.983 | 0.991 | <0.001 |
| Apolipoprotein A | 0.718 | 0.496 | 1.041 | 0.081 | 0.728 | 0.62 | 0.854 | 0.0001 | 0.646 | 0.572 | 0.729 | <0.001 | 0.695 | 0.642 | 0.753 | <0.001 |
| Urate | 0.999 | 0.998 | 1.000 | 0.049 | 0.999 | 0.998 | 0.999 | <0.001 | 0.999 | 0.998 | 0.999 | <0.001 | 0.999 | 0.999 | 0.999 | <0.001 |
| Mean corpuscular volume | 0.971 | 0.954 | 0.988 | 0.001 | 0.982 | 0.974 | 0.989 | <0.001 | 0.98 | 0.975 | 0.986 | <0.001 | 0.988 | 0.984 | 0.992 | <0.001 |
| Urea | 1.046 | 0.988 | 1.107 | 0.123 | 1.041 | 1.014 | 1.068 | 0.002 | 1.043 | 1.022 | 1.063 | <0.001 | 1.037 | 1.023 | 1.051 | <0.001 |
| IGF-1 | 0.997 | 0.982 | 1.012 | 0.693 | 1.003 | 0.997 | 1.009 | 0.367 | 1.004 | 0.999 | 1.009 | 0.136 | 1.004 | 1.001 | 1.008 | 0.007 |
| Glucose | 1.003 | 0.913 | 1.029 | 0.312 | 1.010 | 0.987 | 1.033 | 0.408 | 1.009 | 0.991 | 1.026 | 0.337 | 1.007 | 1.001 | 1.019 | 0.044 |
| Potassium in urine | 0.999 | 0.997 | 1.002 | 0.585 | 1.000 | 0.999 | 1.001 | 0.855 | 1.000 | 0.999 | 1.001 | 0.6 | 0.9995 | 0.999 | 1.0001 | 0.086 |
| Hypertension time | 1.001 | 0.979 | 1.019 | 0.895 | 1.016 | 1.009 | 1.024 | <0.001 | 1.014 | 1.009 | 1.02 | <0.001 | 1.014 | 1.010 | 1.018 | <0.001 |
| Vitamin D | 0.998 | 0.994 | 1.002 | 0.388 | 1.000 | 0.998 | 1.001 | 0.596 | 1.000 | 0.998 | 1.001 | 0.505 | 0.999 | 0.998 | 0.999 | 0.014 |
| SHBG | 0.999 | 0.994 | 1.004 | 0.763 | 0.997 | 0.995 | 0.999 | 0.016 | 0.9983 | 0.9966 | 1.0001 | 0.058 | 0.999 | 0.9979 | 1.0002 | 0.105 |
| Serum creatinine | 1.001 | 0.999 | 1.004 | 0.255 | 1.001 | 0.999 | 1.002 | 0.365 | 1.0001 | 0.9989 | 1.0013 | 0.891 | 1.0006 | 1.0001 | 1.0009 | 0.045 |
| Phosphate | 1.163 | 0.693 | 1.952 | 0.568 | 1.074 | 0.858 | 1.344 | 0.532 | 1.103 | 0.933 | 1.304 | 0.249 | 1.049 | 0.939 | 1.172 | 0.398 |
| Basal metabolic rate | 1.000 | 1.000 | 1.000 | 0.21 | 1.000 | 1.000 | 1.000 | 0.005 | 1.00005 | 1.00002 | 1.00007 | 0.001 | 1.00004 | 1.00002 | 1.00006 | <0.001 |
| Mean platelet thrombocyte volume | 0.950 | 0.883 | 1.022 | 0.171 | 0.975 | 0.945 | 1.007 | 0.12 | 0.977 | 0.954 | 0.999 | 0.046 | 0.986 | 0.971 | 1.001 | 0.075 |
| Miserableness |  |  |  |  |  |  |  |  |  |  |  |  |  |  |  |  |
| No | Ref. |  |  |  | Ref. |  |  |  | Ref. |  |  |  | Ref. |  |  |  |
| Yes | 1.216 | 1.034 | 1.429 | 0.018 | 1.204 | 1.122 | 1.291 | <0.001 | 1.235 | 1.172 | 1.301 | <0.001 | 1.224 | 1.183 | 1.267 | <0.001 |

HR: hazards ratio; LL: lower confidence limit; UL: upper confidence limit; IGF-1: Insulin-Like Growth Factor-1; SHBG: sex hormone binding globulin.

Table S11. Sensitivity analyses for the performance of LightGBM model based on 30 combinations of age and other four predictors.

| Combination | Predictor 1 | Predictor 2 | Predictor 3 | Predictor 4 | AUC | AUC_std |
| --- | --- | --- | --- | --- | --- | --- |
| 1 | Apolipoprotein_A | Vitamin_D | SHBG | Creatinine | 0.679 | 0.004 |
| 2 | Urea | Hypertension_time | Vitamin_D | Phosphate | 0.678 | 0.004 |
| 3 | Urate | Urea | Hypertension_time | Creatinine | 0.678 | 0.005 |
| 4 | Potassium_in_urine | Vitamin_D | Miserableness | Mean_platelet_thrombocyte_volume | 0.679 | 0.004 |
| 5 | Apolipoprotein_A | Vitamin_D | Miserableness | Mean_platelet_thrombocyte_volume | 0.682 | 0.005 |
| 6 | Apolipoprotein_A | IGF_1 | Glucose | Hypertension_time | 0.681 | 0.004 |
| 7 | Mean_corpuscular_volume | Potassium_in_urine | Vitamin_D | Miserableness | 0.681 | 0.004 |
| 8 | Mean_corpuscular_volume | IGF_1 | Potassium_in_urine | Hypertension_time | 0.680 | 0.003 |
| 9 | Apolipoprotein_A | IGF_1 | Creatinine | Miserableness | 0.681 | 0.005 |
| 10 | Mean_corpuscular_volume | Vitamin_D | Creatinine | Basal_metabolic_rate | 0.679 | 0.003 |
| 11 | Total_protein | Mean_corpuscular_volume | Potassium_in_urine | Miserableness | 0.682 | 0.003 |
| 12 | IGF_1 | Phosphate | Miserableness | Basal_metabolic_rate | 0.680 | 0.004 |
| 13 | Total_protein | Urea | Glucose | Mean_platelet_thrombocyte_volume | 0.678 | 0.003 |
| 14 | Urea | IGF_1 | Glucose | Mean_platelet_thrombocyte_volume | 0.677 | 0.004 |
| 15 | Apolipoprotein_A | Urea | Potassium_in_urine | Creatinine | 0.680 | 0.004 |
| 16 | Potassium_in_urine | Vitamin_D | SHBG | Miserableness | 0.680 | 0.005 |
| 17 | Urea | Potassium_in_urine | Creatinine | Mean_platelet_thrombocyte_volume | 0.677 | 0.004 |
| 18 | Urate | SHBG | Phosphate | Miserableness | 0.680 | 0.004 |
| 19 | Urea | SHBG | Creatinine | Miserableness | 0.679 | 0.004 |
| 20 | Urate | Vitamin_D | Phosphate | Basal_metabolic_rate | 0.678 | 0.004 |
| 21 | Apolipoprotein_A | Urea | Glucose | Basal_metabolic_rate | 0.680 | 0.004 |
| 22 | Total_protein | Glucose | Creatinine | Mean_platelet_thrombocyte_volume | 0.677 | 0.003 |
| 23 | Apolipoprotein_A | Urate | Urea | Basal_metabolic_rate | 0.681 | 0.004 |
| 24 | Total_protein | Glucose | Creatinine | Phosphate | 0.677 | 0.004 |
| 25 | Mean_corpuscular_volume | SHBG | Creatinine | Phosphate | 0.679 | 0.003 |
| 26 | Total_protein | Apolipoprotein_A | Urea | Basal_metabolic_rate | 0.680 | 0.004 |
| 27 | Total_protein | Vitamin_D | Phosphate | Mean_platelet_thrombocyte_volume | 0.678 | 0.003 |
| 28 | Apolipoprotein_A | Potassium_in_urine | Vitamin_D | Creatinine | 0.680 | 0.004 |
| 29 | Total_protein | Urate | Hypertension_time | Creatinine | 0.678 | 0.004 |
| 30 | Mean_corpuscular_volume | Hypertension_time | SHBG | Phosphate | 0.680 | 0.004 |

AUC: area under curve; std: standard deviation.

Table S12. External validation based on LightGBM in the CHARLS and Fengshen cohorts.

| **Model** | **AUC** | **95%CI of AUC** | **Sensitivity** | **Specificity** | **PPV** | **NPV** | **Weighted F1-score** |
| --- | --- | --- | --- | --- | --- | --- | --- |
| **CHARLS** |  |  |  |  |  |  |  |
| **all** | 0.647 (0.018) | 0.612,0.682 | 0.797 (0.005) | 0.384 (0.007) | 0.092 (0.004) | 0.960 (0.003) | 0.834 (0.005) |
| **1-year** | 0.612 (0.017) | 0.579,0.645 | 0.676 (0.006) | 0.506 (0.007) | 0.023 (0.002) | 0.989 (0.001) | 0.791 (0.006) |
| **3-year** | 0.654 (0.019) | 0.617,0.691 | 0.680 (0.006) | 0.518 (0.007) | 0.059 (0.003) | 0.973 (0.002) | 0.652 (0.006) |
| **5-year** | 0.647 (0.017) | 0.614,0.680 | 0.712 (0.006) | 0.510 (0.007) | 0.093 (0.004) | 0.962 (0.003) | 0.782 (0.006) |
| **Fengshen** |  |  |  |  |  |  |  |
| **all** | 0.635 (0.031) | 0.574,0.696 | 0.815(0.024) | 0.523(0.031) | 0.162(0.023) | 0.962(0.012) | 0.636(0.029) |
| **1-year** | 0.667 (0.061) | 0.547,0.861 | 0.750 (0.027) | 0.640 (0.027) | 0.082 (0.017) | 0.990 (0.006) | 0.826 (0.023) |
| **3-year** | 0.640 (0.066) | 0.509,0.771 | 0.682(0.032) | 0.554(0.034) | 0.153(0.025) | 0.936(0.017) | 0.649(0.033) |
| **5-year** | 0.622 (0.064) | 0.501,0.747 | 0.682(0.044) | 0.630(0.046) | 0.386(0.046) | 0.853(0.033) | 0.691(0.044) |

AUC: area under curve; PPV: positive predictive value; NPV: negative predictive value.

Values were presented with mean (standard error).

Results were calculated based on the regressed predicted probabilities. Cut-offs were determined based on the achievement of the largest Youden index (YI = sensitivity + specificity - 1).

Table S13. The performance of LightGBM for predicting BPH risk in the UK biobank in different age ranges with 17 variables.

| **Model** | **Age range** | **AUC** | **Sensitivity** | **Specificity** | **PPV** | **NPV** | **Weighted F1-score** |
| --- | --- | --- | --- | --- | --- | --- | --- |
| **all** | 45 years old and above | 0.688±0.002 | 0.722±0.009 | 0.511±0.008 | 0.138±0.002 | 0.944±0.002 | 0.620±0.005 |
|  | 50 years old and above | 0.654±0.006 | 0.696±0.013 | 0.485±0.005 | 0.144±0.002 | 0.928±0.002 | 0.593±0.003 |
|  | 55 years old and above | 0.622±0.008 | 0.568±0.006 | 0.561±0.008 | 0.156±0.002 | 0.901±0.002 | 0.636±0.006 |
|  | 60 years old and above | 0.602±0.007 | 0.603±0.029 | 0.495±0.022 | 0.161±0.005 | 0.886±0.005 | 0.582±0.014 |
| **1-year** | 45 years old and above | 0.687±0.003 | 0.653±0.077 | 0.540±0.085 | 0.016±0.002 | 0.993±0.001 | 0.689±0.070 |
|  | 50 years old and above | 0.653±0.004 | 0.635±0.020 | 0.555±0.013 | 0.018±0.001 | 0.992±0.001 | 0.703±0.010 |
|  | 55 years old and above | 0.622±0.003 | 0.639±0.038 | 0.515±0.032 | 0.019±0.001 | 0.990±0.001 | 0.667±0.027 |
|  | 60 years old and above | 0.600±0.003 | 0.632±0.046 | 0.484±0.032 | 0.020±0.001 | 0.987±0.001 | 0.639±0.028 |
| **3-year** | 45 years old and above | 0.688±0.004 | 0.745±0.019 | 0.520±0.012 | 0.028±0.001 | 0.991±0.001 | 0.670±0.010 |
|  | 50 years old and above | 0.656±0.002 | 0.751±0.019 | 0.445±0.011 | 0.028±0.001 | 0.988±0.001 | 0.602±0.010 |
|  | 55 years old and above | 0.621±0.003 | 0.609±0.019 | 0.532±0.017 | 0.032±0.001 | 0.982±0.001 | 0.675±0.014 |
|  | 60 years old and above | 0.603±0.003 | 0.561±0.017 | 0.539±0.026 | 0.034±0.002 | 0.977±0.001 | 0.677±0.021 |
| **5-year** | 45 years old and above | 0.691±0.005 | 0.750±0.019 | 0.509±0.009 | 0.050±0.002 | 0.983±0.002 | 0.651±0.008 |
|  | 50 years old and above | 0.661±0.004 | 0.738±0.011 | 0.459±0.006 | 0.051±0.001 | 0.978±0.001 | 0.604±0.005 |
|  | 55 years old and above | 0.625±0.003 | 0.540±0.031 | 0.606±0.026 | 0.059±0.003 | 0.966±0.001 | 0.716±0.018 |
|  | 60 years old and above | 0.605±0.006 | 0.587±0.019 | 0.508±0.019 | 0.060±0.002 | 0.958±0.001 | 0.636±0.016 |
| **10-year** | 45 years old and above | 0.691±0.005 | 0.686±0.028 | 0.568±0.018 | 0.120±0.005 | 0.955±0.004 | 0.672±0.011 |
|  | 50 years old and above | 0.667±0.006 | 0.670±0.015 | 0.527±0.005 | 0.122±0.001 | 0.942±0.003 | 0.633±0.002 |
|  | 55 years old and above | 0.635±0.003 | 0.675±0.007 | 0.469±0.011 | 0.127±0.003 | 0.926±0.001 | 0.580±0.009 |
|  | 60 years old and above | 0.610±0.003 | 0.518±0.035 | 0.593±0.024 | 0.145±0.003 | 0.903±0.005 | 0.658±0.013 |

AUC: area under curve; PPV: positive predictive value; NPV: negative predictive value.

Results were calculated based on the regressed predicted probabilities. Cut-offs were determined based on the achievement of the largest Youden index (YI = sensitivity + specificity - 1).

Table S14. The performance of LightGBM for predicting BPH risk in the UK biobank in different age ranges with 5 variables.

| **Model** | **Age range** | **AUC** | **Sensitivity** | **Specificity** | **PPV** | **NPV** | **Weighted F1-score** |
| --- | --- | --- | --- | --- | --- | --- | --- |
| **all** | 45 years old and above | 0.671±0.013 | 0.727±0.013 | 0.495±0.010 | 0.136±0.002 | 0.943±0.002 | 0.608±0.007 |
|  | 50 years old and above | 0.650±0.004 | 0.713±0.004 | 0.450±0.009 | 0.139±0.002 | 0.927±0.001 | 0.565±0.008 |
|  | 55 years old and above | 0.555±0.022 | 0.545±0.025 | 0.562±0.024 | 0.150±0.002 | 0.897±0.002 | 0.634±0.016 |
|  | 60 years old and above | 0.597±0.012 | 0.587±0.012 | 0.488±0.017 | 0.156±0.005 | 0.880±0.004 | 0.575±0.013 |
| **1-year** | 45 years old and above | 0.661±0.014 | 0.640±0.103 | 0.535±0.079 | 0.015±0.001 | 0.993±0.001 | 0.684±0.065 |
|  | 50 years old and above | 0.645±0.015 | 0.648±0.024 | 0.515±0.026 | 0.017±0.001 | 0.991±0.001 | 0.669±0.022 |
|  | 55 years old and above | 0.613±0.003 | 0.505±0.049 | 0.615±0.026 | 0.019±0.001 | 0.988±0.001 | 0.747±0.018 |
|  | 60 years old and above | 0.582±0.032 | 0.595±0.070 | 0.468±0.070 | 0.019±0.001 | 0.986±0.001 | 0.621±0.066 |
| **3-year** | 45 years old and above | 0.667±0.009 | 0.797±0.011 | 0.459±0.007 | 0.027±0.001 | 0.992±0.001 | 0.617±0.006 |
|  | 50 years old and above | 0.654±0.004 | 0.808±0.015 | 0.372±0.010 | 0.027±0.001 | 0.989±0.001 | 0.531±0.010 |
|  | 55 years old and above | 0.616±0.003 | 0.538±0.025 | 0.582±0.025 | 0.031±0.001 | 0.981±0.001 | 0.714±0.019 |
|  | 60 years old and above | 0.581±0.005 | 0.498±0.031 | 0.571±0.027 | 0.033±0.001 | 0.975±0.001 | 0.701±0.021 |
| **5-year** | 45 years old and above | 0.667±0.005 | 0.787±0.022 | 0.460±0.010 | 0.048±0.002 | 0.984±0.002 | 0.609±0.008 |
|  | 50 years old and above | 0.656±0.003 | 0.655±0.024 | 0.530±0.023 | 0.052±0.001 | 0.975±0.001 | 0.664±0.018 |
|  | 55 years old and above | 0.616±0.003 | 0.563±0.024 | 0.564±0.015 | 0.056±0.002 | 0.966±0.002 | 0.685±0.011 |
|  | 60 years old and above | 0.582±0.003 | 0.588±0.021 | 0.484±0.022 | 0.057±0.003 | 0.957±0.002 | 0.615±0.019 |
| **10-year** | 45 years old and above | 0.664±0.006 | 0.724±0.022 | 0.520±0.017 | 0.115±0.005 | 0.956±0.004 | 0.636±0.012 |
|  | 50 years old and above | 0.657±0.008 | 0.724±0.012 | 0.459±0.015 | 0.117±0.002 | 0.944±0.002 | 0.580±0.013 |
|  | 55 years old and above | 0.614±0.007 | 0.537±0.019 | 0.593±0.015 | 0.131±0.004 | 0.918±0.002 | 0.668±0.010 |
|  | 60 years old and above | 0.595±0.004 | 0.537±0.021 | 0.558±0.018 | 0.139±0.003 | 0.901±0.003 | 0.634±0.011 |

AUC: area under curve; PPV: positive predictive value; NPV: negative predictive value.

Results were calculated based on the regressed predicted probabilities. Cut-offs were determined based on the achievement of the largest Youden index (YI = sensitivity + specificity - 1).


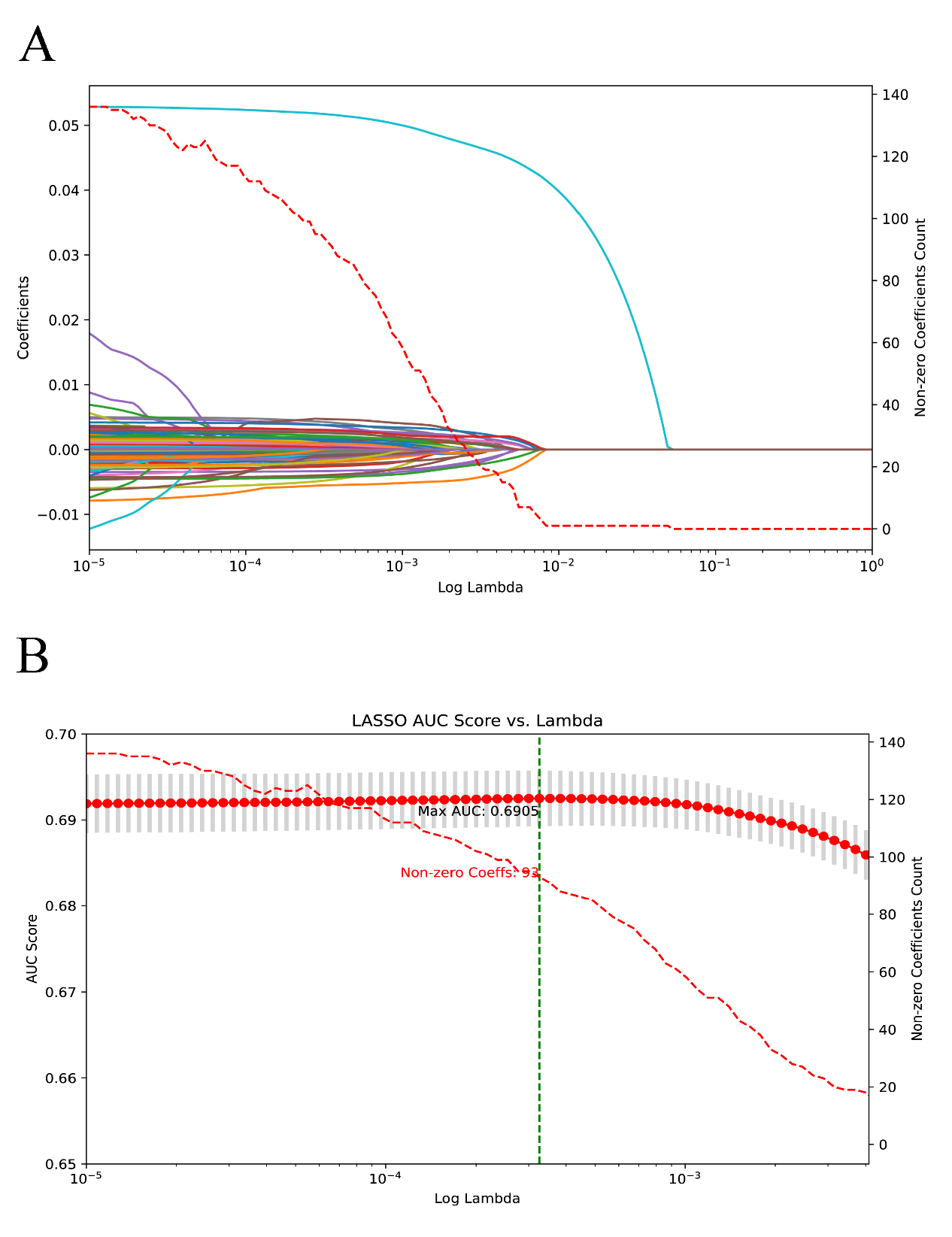


Fig. S1. Predictors screened by LASSO in the UK biobank.

(A) Predictor screening process; (B) Log lambda corresponds to the best AUC and the number of predictors of non-zero coefficients.


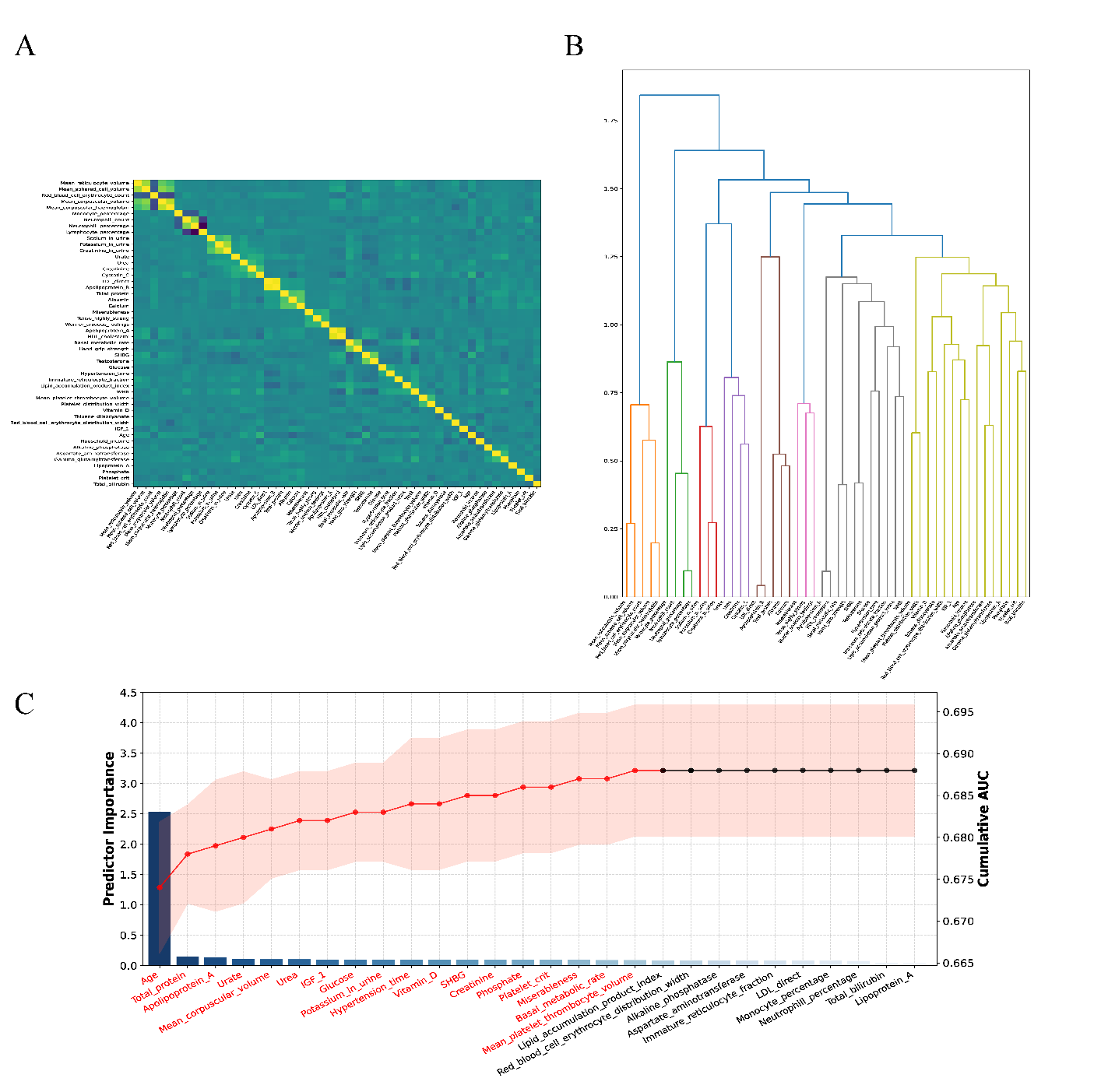


Fig. S2. Predictors screened by Light GBM in UK biobank.

(A) Heatmap of correlations between predictors; (B) Hierarchical clustering based on correlation analysis; (C) Sequential forward selection for predictor screening.


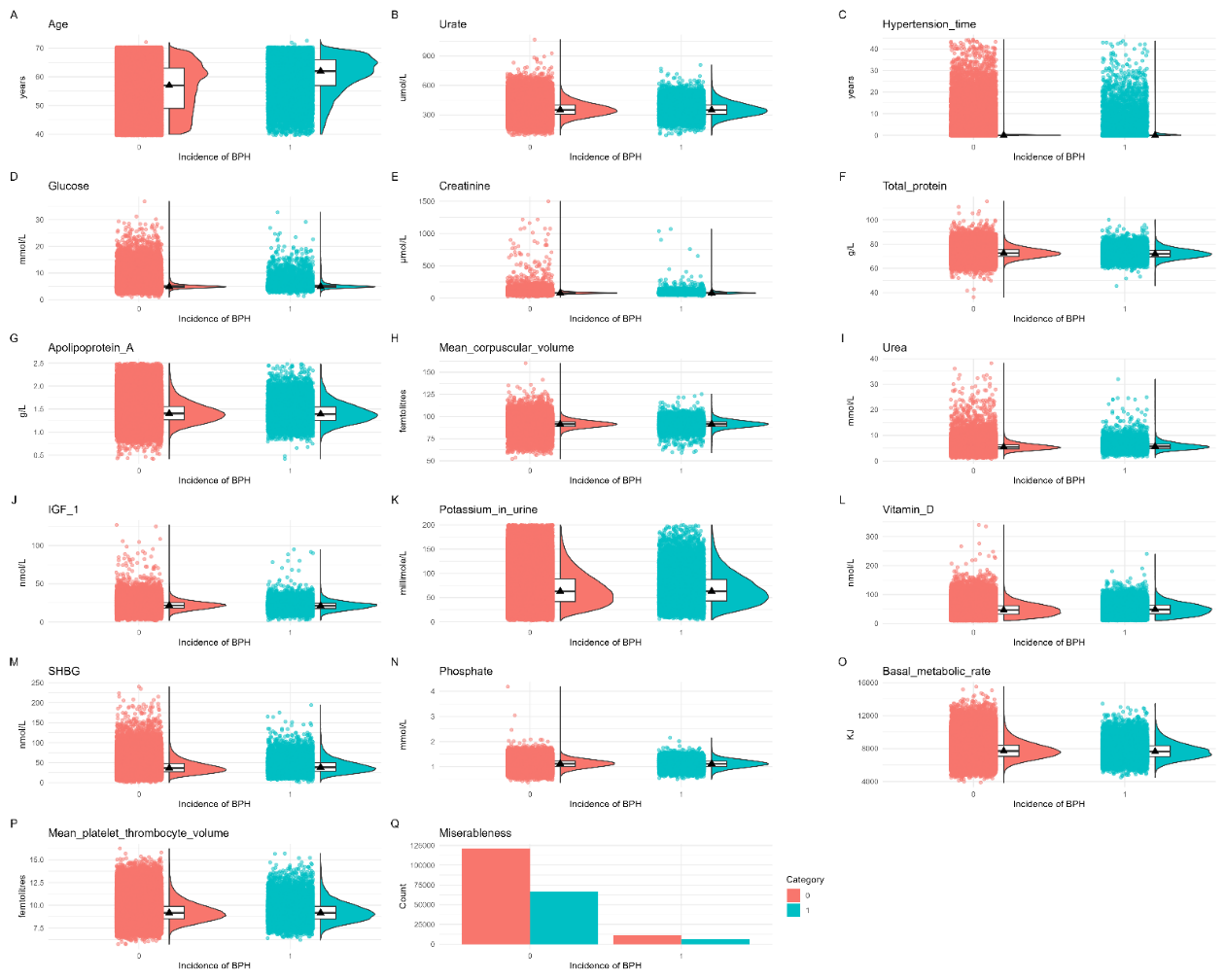


Fig. S3. Violin plots of 17 variables between participants with BPH and without BPH in the UK Biobank.


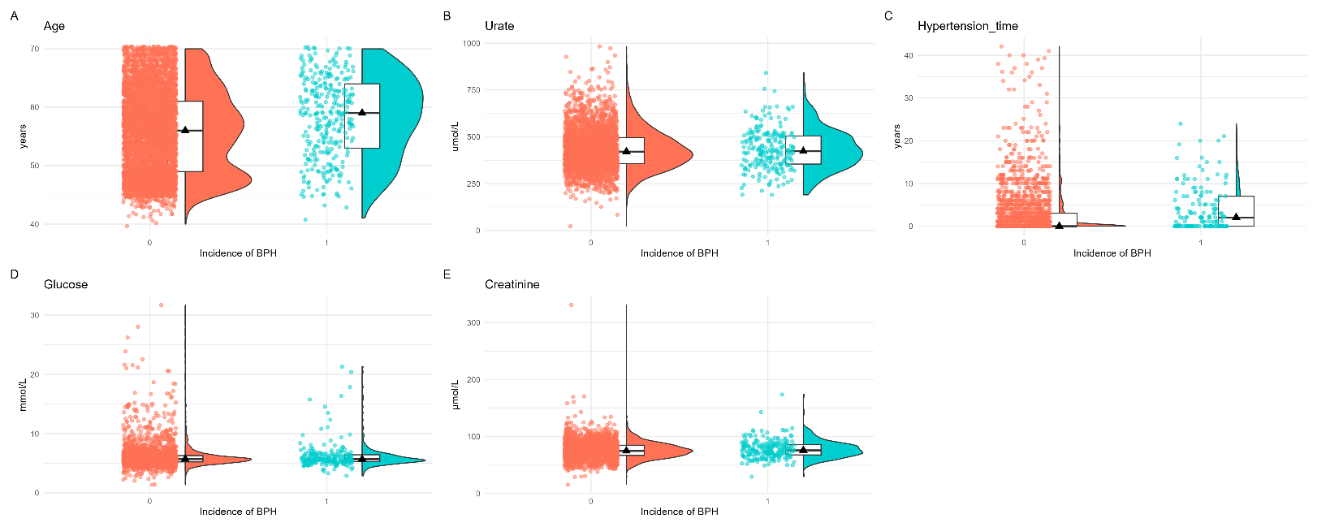


Fig. S4. Violin plots of 5 variables between participants with BPH and without BPH in the CHARLS.


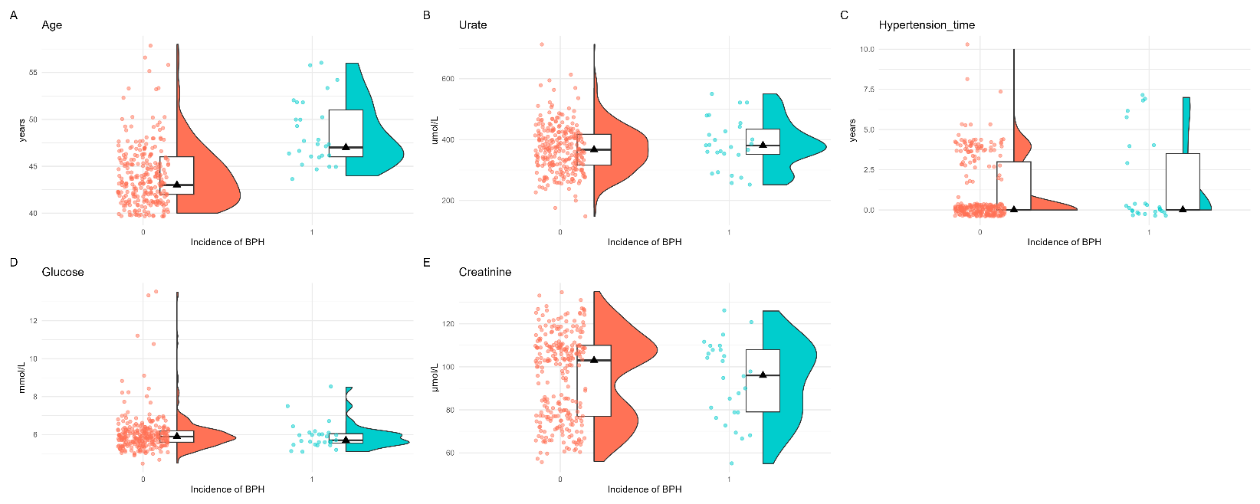


Fig. S5. Violin plots of 5 variables between participants with BPH and without BPH in the Fengshen study.


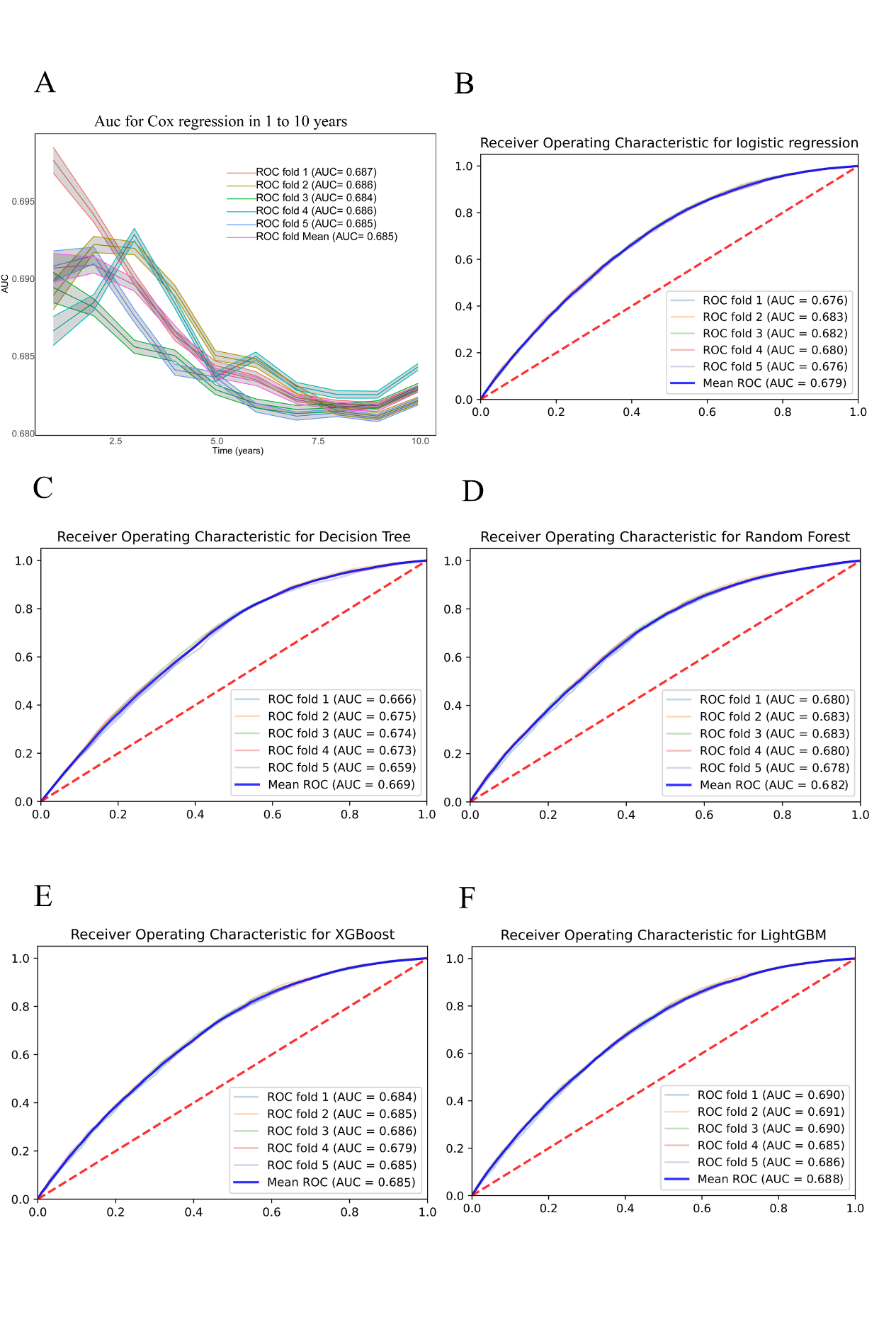


Fig. S6. AUC plots of the full model based on different methods on all incident BPH in the UK biobank.

(A) Cox regression; (B) Logistic regression; (C) Decision tree; (D) Random forest; (E) XGBoost; (F) LightGBM.


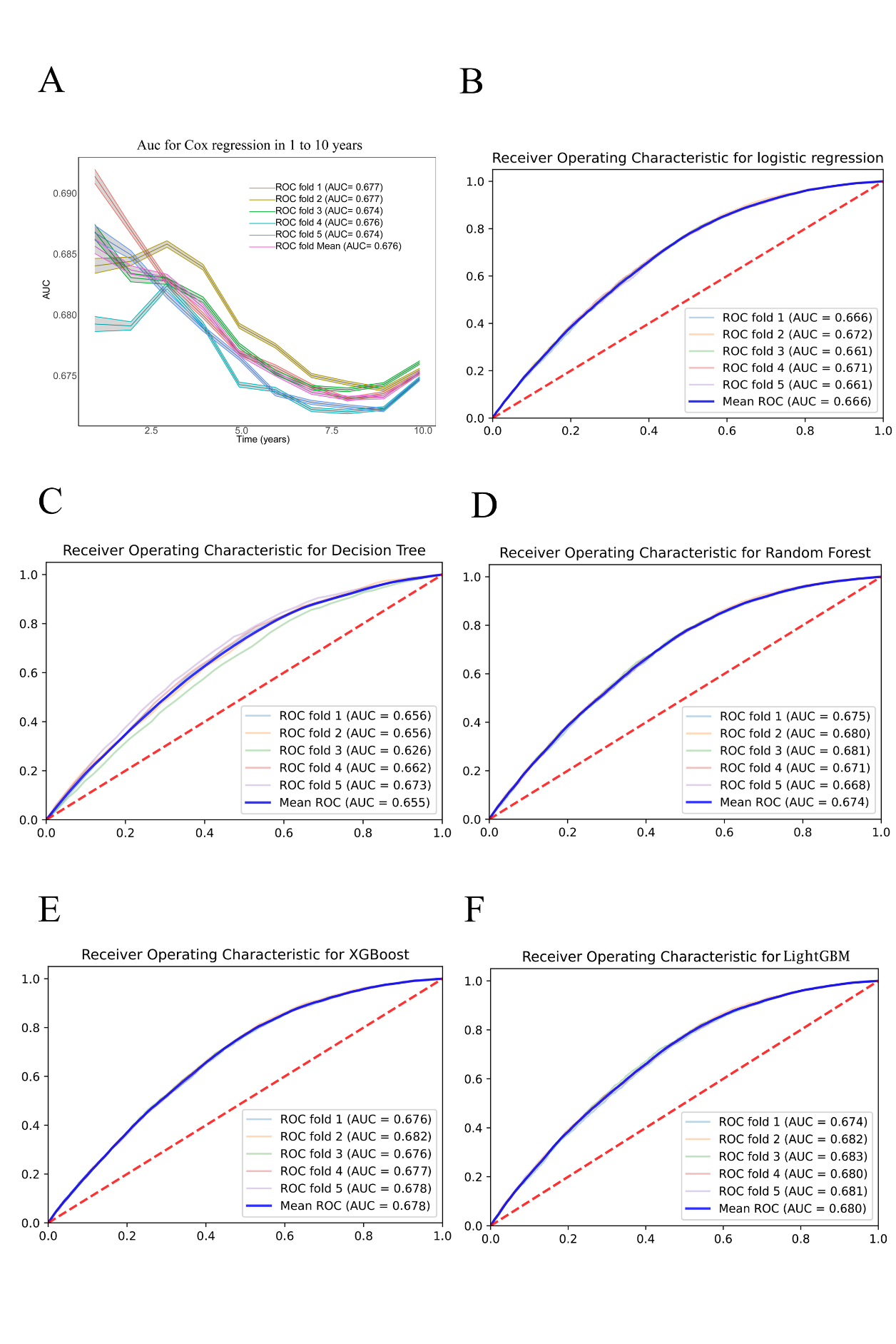


Fig. S7. AUC plots of the simplified model based on different methods on all incident BPH in the UK biobank.

(A) Cox regression; (B) Logistic regression; (C) Decision tree; (D) Random forest; (E) XGBoost; (F) LightGBM.


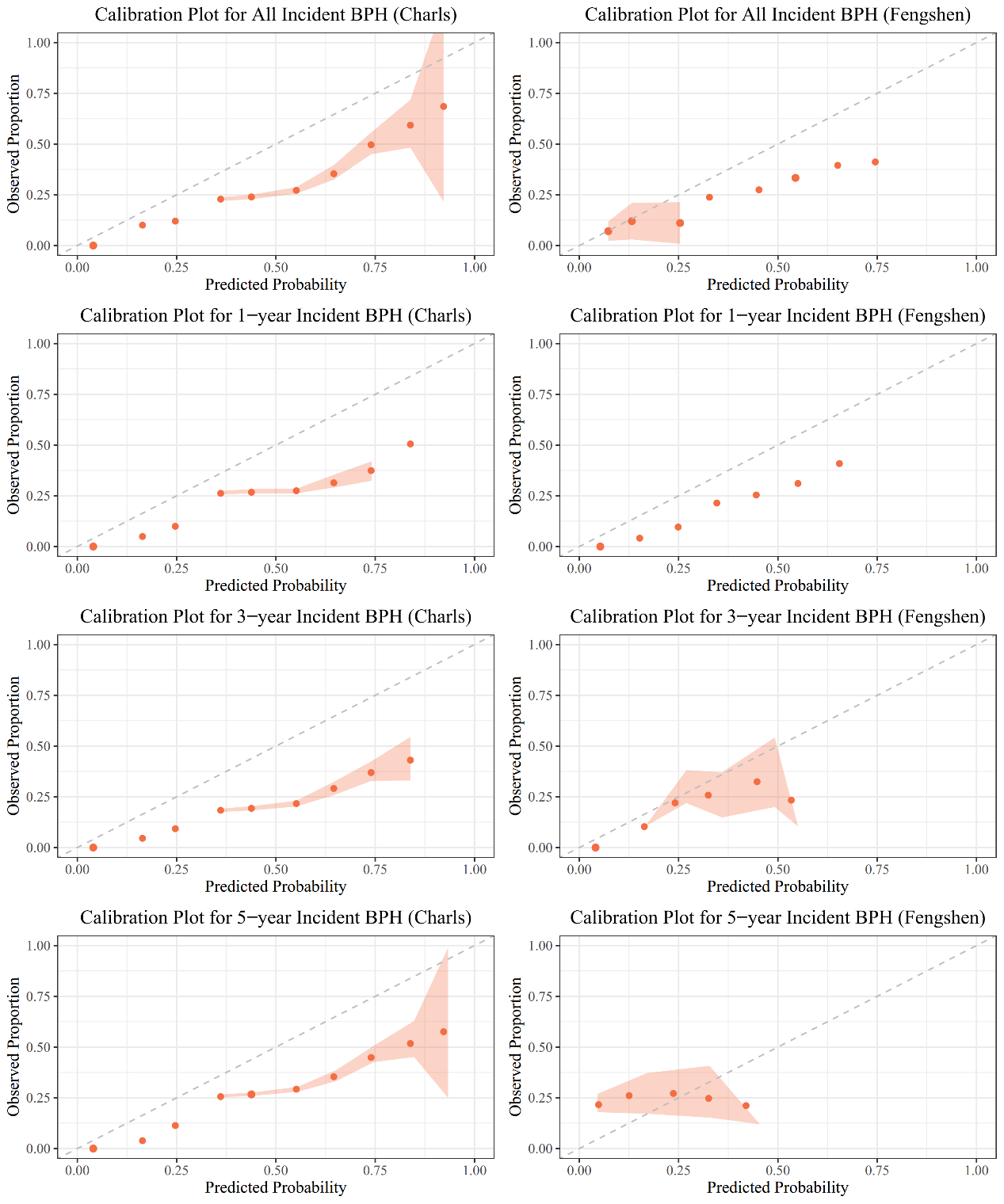


**Fig. S8.** Calibration curve plots of modelling for the simplified model based on LightGBM for predicting all, 1-year, 3-, and 5-year incidence risks in the CHARLS and Fengshen study.

Left panels for CHARLS; Right panels for Fengshen study.


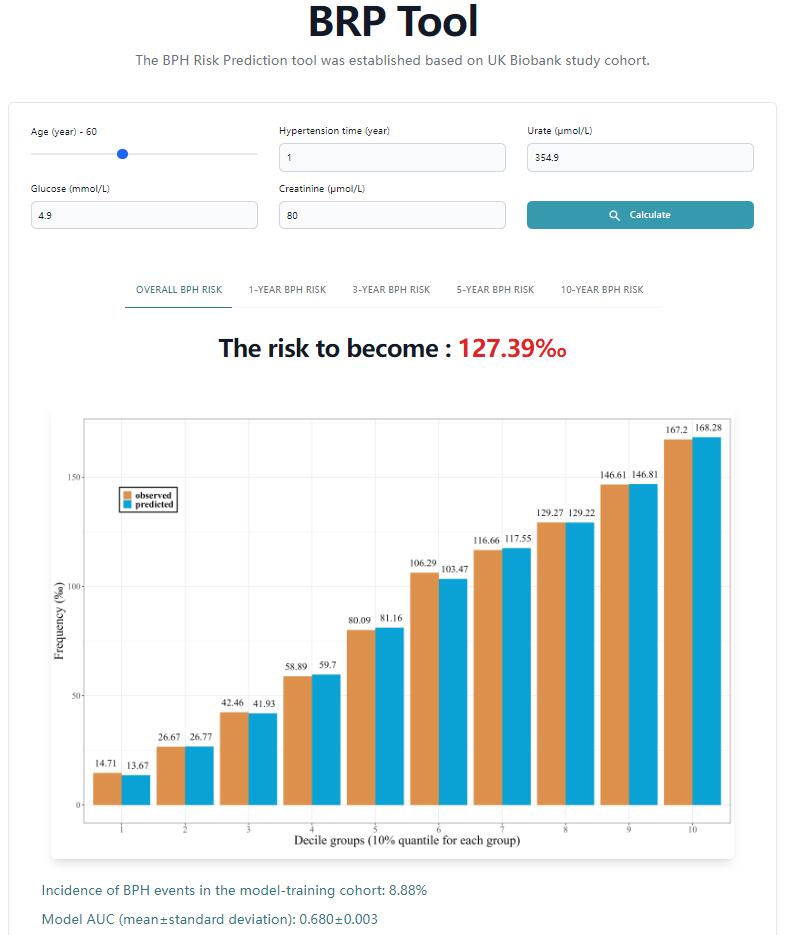


Fig. S9. A stylized representation of the tool.
